# Vaccination protects against COVID-associated pulmonary fibrin deposition

**DOI:** 10.1128/jvi.00633-25

**Published:** 2025-11-06

**Authors:** Joanna Ireland, David Myers, Chang Huang, Cameron Allen, Gwynne Roth, Zhongcheng Zou, Ming Zhao, Motoshi Suzuki, Lisa Olano, Joshua Tan, Shreya M. Kanth, Julio A. Huapaya, Homer Twigg, Anthony F. Suffredini, Peter Sun

**Affiliations:** 1 Laboratory of Immunogenetics, National Institute of Allergy and Infectious Diseases, National Institutes of Health, Rockville, Maryland, USA; 2 Research Technologies Branch, National Institute of Allergy and Infectious Diseases, National Institutes of Health, Rockville, Maryland, USA; 3 Critical Care Medicine Branch, National Heart, Lung, and Blood Institute, National Institutes of Health, Bethesda, Maryland, USA; 4 Critical Care Medicine Department, Clinical Center, National Institutes of Health 24481, Bethesda, Maryland, USA; 5 Division of Pulmonary, Critical Care, Sleep, and Occupational Medicine, Indiana University Medical Center, Indianapolis, Indiana, USA; University of North Carolina at Chapel Hill, Chapel Hill, North Carolina, USA

**Keywords:** COVID-19 vaccination, SARS-CoV-2 infection, BALF proteomics, coagulation, fibrin deposition

## Abstract

Understanding the protective mechanism of severe acute respiratory syndrome coronavirus 2 (SARS-CoV-2) vaccines against severe COVID is important for therapeutic development to mitigate disease-associated lung pathologies. Here, we investigated the association between vaccination and the disease severity with a particular emphasis on viral-induced pulmonary fibrin formation in 43 COVID individuals. While COVID vaccination reduced the disease severity in this cohort, their plasma coagulation indices, including prothrombin time (PT), partial thromboplastin time (PTT), and D-dimer concentrations, remain unchanged between the vaccinated and non-vaccinated individuals. In contrast, vaccination lowered pulmonary inflammatory and coagulation signatures, reduced fibrinogen concentrations, and prevented prothrombin activation in bronchoalveolar lavage fluid (BALF), such that no viral-induced fibrin was observed in the vaccinated BALF. The formation of viral-induced fibrin correlated with the disease severity and was observed in non-vaccinated BALF samples containing high concentrations of fibrinogen and prothrombin, suggesting vaccination protected against the viral-induced pulmonary fibrin formation. Our finding highlights the use of pulmonary rather than plasma fibrinogen levels as a risk indicator for severe COVID disease.

**IMPORTANCE:**

Understanding the protective mechanism of COVID-19 vaccines against the severity of the disease is important for therapeutic development, and thus, subject to intense investigation. Here, we studied a cohort of 43 COVID patients based on their vaccination status. We showed that (i) COVID disease severity is associated with the formation of SARS-CoV-2-induced pulmonary fibrin, (ii) vaccination protected against severe COVID disease by reducing infiltration of coagulants, preventing prothrombin activation and fibrin deposition in infected lungs, and (iii) plasma coagulation indices are not useful indicators for fibrin deposition in infected lungs. Rather, the level of pulmonary fibrinogen provides an informative indicator for COVID-associated coagulation in lung.

## INTRODUCTION

The rapid developments of vaccines and antiviral drugs contributed to the end of the COVID-19 pandemic (1, 2). Despite frequent occurrences of breakthrough infections (3–5), COVID-19 vaccination reduced the disease severity and protected against mortality (4, 6–10). Severe acute respiratory syndrome coronavirus 2 (SARS-CoV-2) infection can initiate rampant immune responses leading to cytokine storms that are believed to be responsible for severe COVID-associated lung pathology. COVID vaccine consistently reduced inflammation in breakthrough infections (11–14), suggesting vaccination protects against the development of immunopathology resulting from overt inflammatory responses in severe COVID diseases. However, the clinical benefit of therapeutic use of anti-inflammatory compounds was marginal for treating severe COVID (15–18). In addition, vaccination also reduced the disease severity in immunocompromised individuals (19–22), suggesting the benefit of vaccination extends beyond the reduction of inflammation. The potential contributing factors to severe COVID-associated pathophysiology are likely multifaceted, involving inflammatory neutrophil and T cell infiltrations, potential formation of neutrophil extracellular traps, and the presence of other comorbidity factors, such as immunodeficiency and diabetes (23). Early autopsies showed the presence of characteristic fibrin-rich hyaline membranes associated with diffuse alveolar damage (DAD) in infected lungs as a hallmark of severe COVID-19 infections (24–27). D-dimers are degradation fragments of fibrin and are used for assessing the risk of venous thromboembolism (28–30). While some studies support a correlation between higher plasma D-dimer levels and the severity of COVID-19 disease (31–34), others did not (35–39). Furthermore, the therapeutic use of anticoagulant heparin failed to reverse the disease in critically ill patients (16).

**Fig 1:**
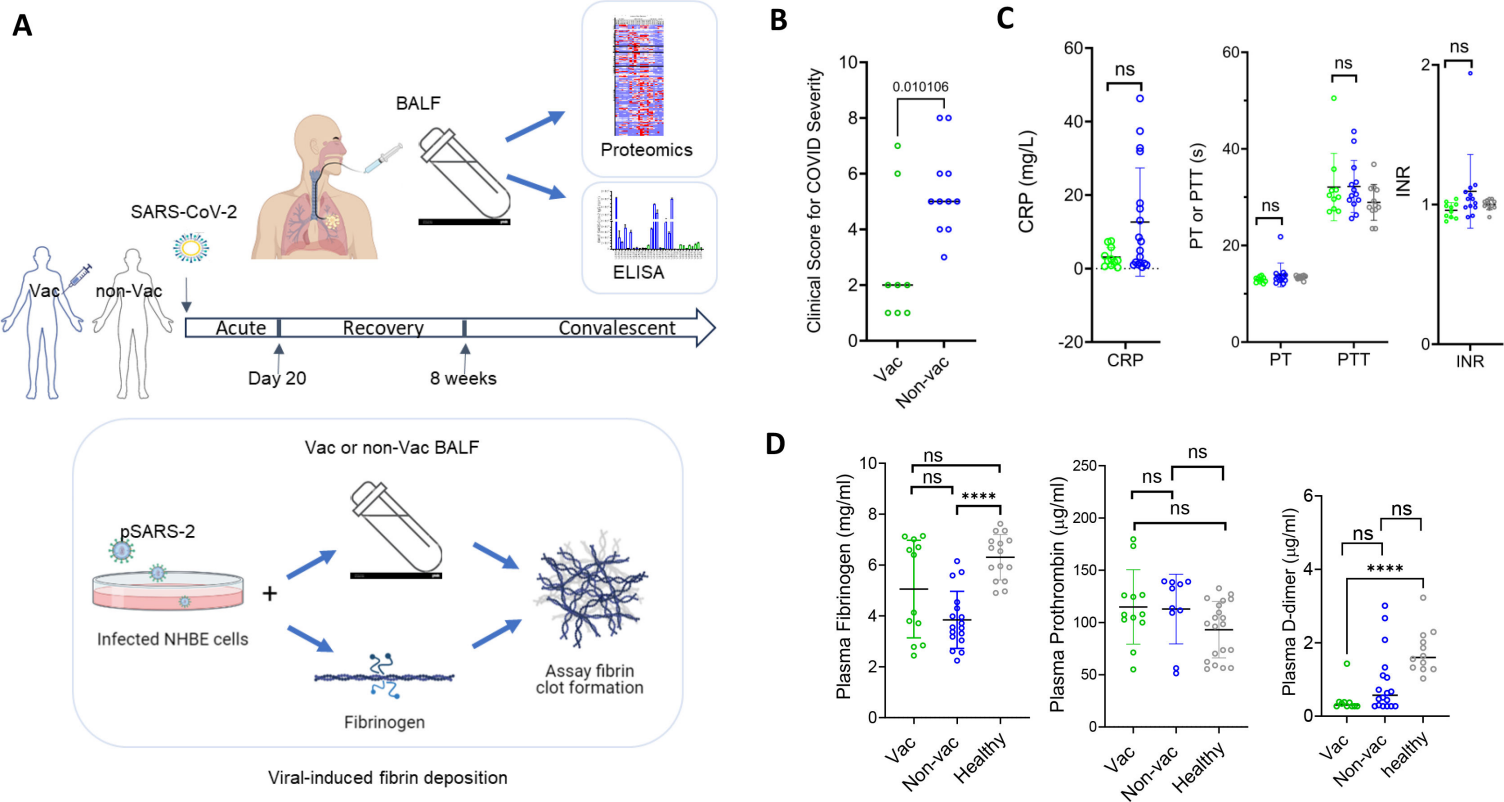
The overall experimental design. (**A**) Schematics for the experimental design. BALF samples from vaccinated and non-vaccinated COVID individuals were taken during acute, recovery, and convalescent phases for proteomics analyses, measurement of coagulation factor concentrations by ELISA, and for the assessment of severe COVID-associated pulmonary fibrin deposition using a viral-induced fibrin clotting model. (**B**) Clinical score for COVID severity (see Materials and Methods for score definition). (**C**) Clinical measurement of prothrombin time (PT), partial thromboplastin time (PTT), international normalized ratio (INR), and CRP concentrations from vaccinated (green), non-vaccinated (blue), and healthy (gray) plasmas. (**D**) Concentrations of fibrinogen, prothrombin, and D-dimer in vaccinated (vac, green circles), non-vaccinated (non-vac, blue circles), or healthy (gray symbols) plasmas were measured by ELISA. Unless noted otherwise, the statistical analyses in all figures were performed using two-tailed unpaired Student’s t test. The *P* values in all figures are indicated as ns >0.05, *<0.05, **<0.01, ***<0.001, ****<0.0001.

We recently described a SARS-CoV-2 infection-induced non-classical coagulation that occurred in pulmonary tissue outside of plasma (40). The viral infection activates the TMPRSS family of serine proteases, such as ST14 and HAT, on lung epithelial cells, leading to prothrombin activation and fibrin deposition in the presence of infiltrating fibrinogen. Here, we investigated the link between COVID vaccination and the viral-induced fibrin deposition. Using bronchoalveolar lavage fluid (BALF) and plasma collected from COVID clinical studies during the early pandemic from 2020 to 2021, we investigated the impact of vaccination on the level of coagulation factors in plasma and pulmonary airways and assessed the risk of viral-induced pulmonary fibrin deposition (Fig. 1A). Our findings clarified the conflicting data regarding the use of D-dimers as the indicator for severe COVID and showed pulmonary fibrinogen concentrations correlated with viral-induced pulmonary fibrin depositions. These findings suggest a new mechanism for vaccination-associated protection against disease severity.

## RESULTS

### Vaccination reduced COVID disease severity

Existing data support the benefit of vaccination in protection against severe COVID diseases despite the occurrence of breakthrough infections (6, 7, 9). To investigate the potential protective mechanisms of COVID vaccines, we investigated the risk of coagulation in plasma and lung among a group of 43 vaccinated and non-vaccinated COVID patients during the early pandemic between 2020 and 2021. Among them, 19 individuals received either mRNA-1273 (Moderna), BNT162b2 (Pfizer), or Ad26.COV2.S (Johnson & Johnson) vaccines and are regarded as having breakthrough infections. The remainder did not receive prior vaccinations. Plasma and bronchoalveolar lavage (BAL) samples were collected to assess the risk of coagulations in blood and pulmonary space, respectively. The samples were further grouped into acute, recovery, and convalescent categories if they were collected within the first 20 days, between 3 and 8 weeks, or after 8 weeks of COVID symptom onset (Fig. 1A). Among acute COVID patients of this study group, vaccination lessened the clinical disease severity (Fig. 1B), consistent with the protective effect of vaccination against COVID severity.

### Plasma coagulation indices did not differ with vaccination status

As coagulopathy is associated with severe COVID-19 (26, 41), it is conceivable that vaccination may lower the risk of hypercoagulation associated with COVID-19. Blood coagulation occurs through extrinsic and intrinsic coagulation pathways, and its risk is measured by prothrombin time (PT) and partial thromboplastin time (PTT) (42–44). Interestingly, despite heightened inflammation in the non-vaccinated group as evidenced by the presence of higher levels of C-reactive protein (CRP) and proinflammatory cytokine (Fig. 1C; Fig. S1A), their plasma coagulation indices, PT and PTT, remained similar to those from vaccinated COVID and healthy plasma samples (Fig. 1C), suggesting inflammation did not affect their plasma coagulation indices. We further measured fibrinogen and prothrombin concentrations in vaccinated and non-vaccinated COVID plasma, as well as in healthy controls by enzyme-linked immunosorbent assay (ELISA). Individual fibrinogen varied between 2 and 8 mg/mL, and prothrombin varied between 50 and 150 µg/mL in plasma samples (Fig. 1D). Overall, the concentrations of fibrinogen, prothrombin, and D-dimer remained similar between vaccinated and non-vaccinated COVID plasma samples (Fig. 1D), consistent with the observations by others (45). Thus, vaccination did not affect coagulation in the blood circulation.

### Vaccination reduced pulmonary immune activation and coagulation signatures

**Fig 2:**
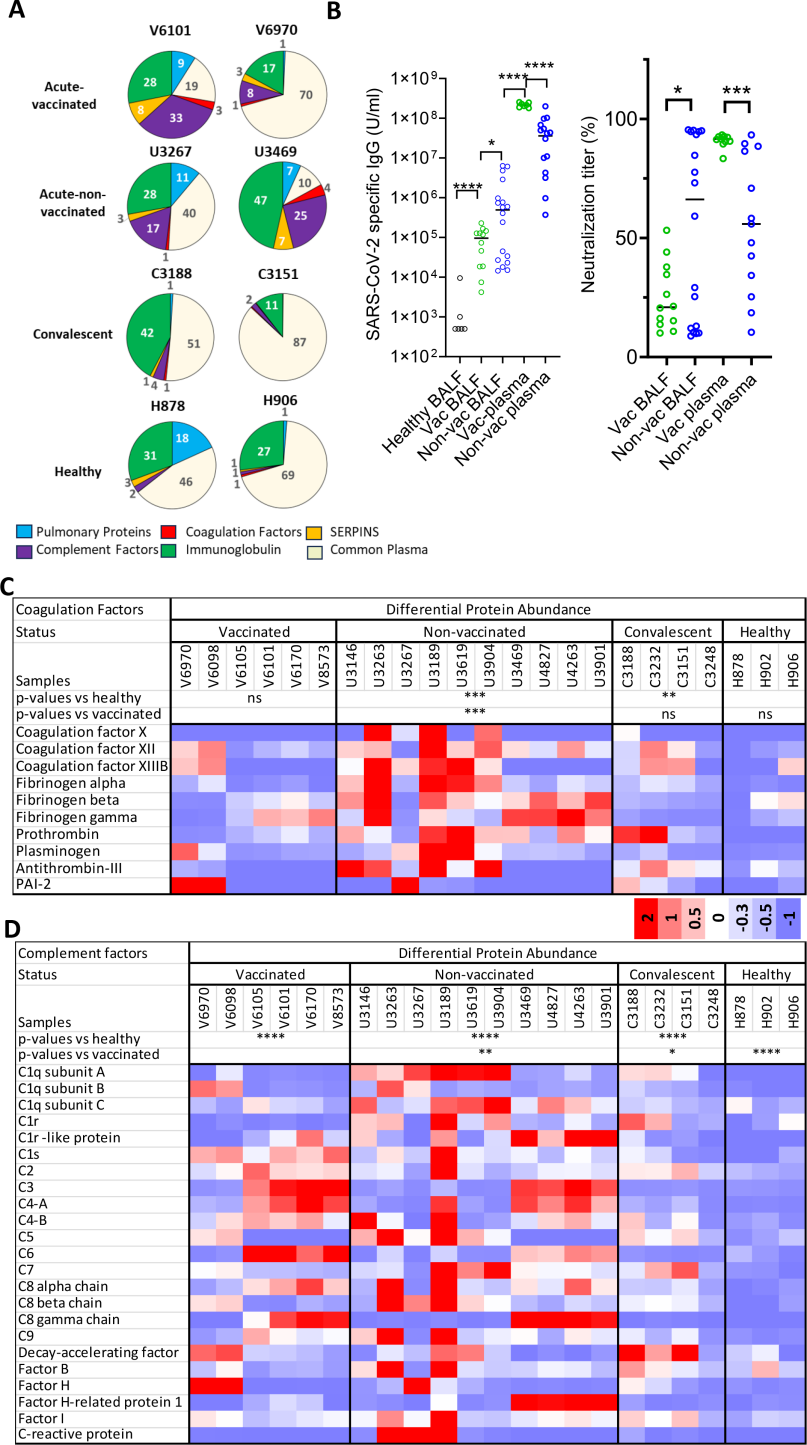
Proteomic analyses of acute (vaccinated, non-vaccinated), convalescent, and healthy BALF. (**A**) Relative abundance of different categories of plasma proteins found in acute (vaccinated, non-vaccinated) and convalescent COVID, as well as healthy BALF. (**B**) The presence of SARS-CoV-2 specific IgG (left panel) and neutralization antibodies (right panel) in vaccinated and non-vaccinated BALF, as well as plasmas. (**C–D**) Heat map showing differential abundances of each category of proteins between acute (vaccinated, non-vaccinated), convalescent COVID, and healthy BALF samples. List of coagulation factors (**C**) and complement factors (**D**) detected in various BALF. Statistical analyses were performed using two-way analysis of variance (ANOVA) between columns. *P < 0.05, **P < 0.01, ***P < 0.001, ****P < 0.0001; ns, not significant.

Despite clear serological differences in both cellular immune responses and inflammatory cytokine activations between vaccinated and non-vaccinated populations (Fig. S1A) (11–13), plasma coagulation parameters PT and PTT did not differ between the two groups (Fig. 1C and D). As viral-induced fibrin deposition does not require the initiation of the extrinsic coagulation pathway in plasma, this prompted us to investigate if pulmonary tissue rather than plasma concentration of coagulation factors is a better indicator for COVID-associated fibrin deposition. Previous proteomic characterizations of BALF from SARS-CoV-2-infected lungs primarily focused on dysregulation of immune function and inflammatory signatures associated with COVID-19 disease severity (46–50). To address the effect of vaccination on fibrinogenic profiles in COVID lung fluids, we performed proteomic analyses on BAL fluids (BALF) by mass spectrometry from 6 vaccinated, 10 non-vaccinated (both acute and recovery) COVID individuals, as well as 4 convalescent COVID and two healthy individuals. In total, mass spectrometry identified approximately 1,000 common proteins from these BALF samples (Table S1). Approximately 200 proteins are pulmonary and plasma proteins, including surfactants and mucins, common enriched plasma proteins, coagulation factors, complement factors, serpins, and immunoglobulins (Table S1; Fig. 2A; Fig. S1B). The rest are intracellular proteins, presumably released from apoptotic cells. Among infiltrated plasma proteins, the most abundant ones are from common enriched plasma proteins, immunoglobulins, complement factors, and serpins categories, indicating a significant presence of both adaptive and innate immune systems in lung. Differential protein abundance analysis showed an increased abundance of complement components in all COVID BALF, including vaccinated, non-vaccinated, and convalescent COVID samples compared to the healthy controls, consistent with the activation of innate immune responses to SARS-CoV-2 infections (Fig. 2D; Fig. S2A). The presence of more abundant immunoglobulins in the non-vaccinated than the vaccinated lung fluids is consistent with a recent finding of non-vaccinated BALF forming clusters containing more inflammatory proteins than vaccinated BALF (Fig. 2A; Fig. S2A and B) (46). Given that immunizations in general increase antigen-specific antibody titers, we further measured the concentration of SARS-CoV-2 spike-specific IgG, as well as the neutralization antibody titers in non-vaccinated and vaccinated samples (Fig. 2B; Fig. S3). As expected, higher levels of SARS-CoV-2 specific IgGs are present in vaccinated than in the non-vaccinated plasma samples. To the contrary, the order of SARS-CoV-2 specific antibodies levels, as well as their neutralization antibody titers in BALF, is reversed between the vaccinated and non-vaccinated individuals (Fig. 2B). The result is counterintuitive and suggests a decreased plasma infiltration in vaccinated lungs.

### Vaccination reduced pulmonary fibrinogen, prothrombin, and D-dimer concentrations

**Fig 3:**
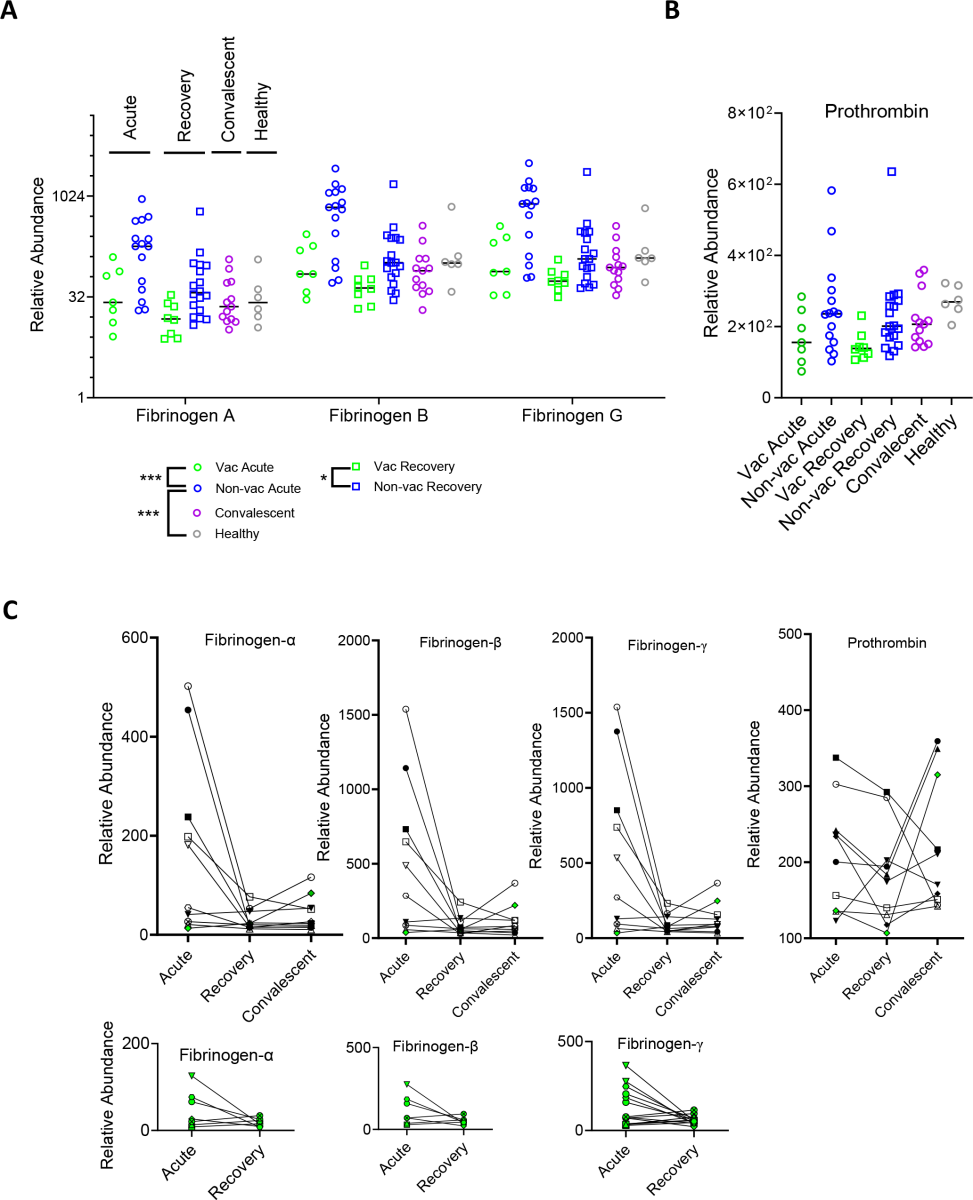
Proteomic analysis of fibrinogen and prothrombin abundance in various BALF samples. Relative abundance of fibrinogen-α, -β, -γ (**A**) and prothrombin (**B**) in 7 vaccinated (green circle) and 15 non-vaccinated (blue circle) acute COVID (1–40 days of symptom onset), 8 vaccinated (green square) and 18 non-vaccinated (blue square) recovery (41–84 days), as well as 13 convalescent (84 days–13 month, purple circle) and 6 healthy BALF samples as determined by data-independent acquisition mass spectrometry (DIA-MS) (46). Statistics using abundances from all three fibrinogen subunits and prothrombin by two-way ANOVA, resulting in *P* values *** (<0.001) between vac and non-vac, between non-vac and healthy, ns between vac and healthy, and ns between convalescent and healthy groups. (**C**) Mass spectrometry determination of the relative abundances of fibrinogen-αβγ and prothrombin in acute, recovery, and convalescent BALF samples from non-vaccinated (top) and vaccinated (bottom, green symbols) individuals who are separated by different symbols. The majority of the vaccinated BALF samples, except one (green symbols on the top panel), were collected for acute and recovery but not convalescent phases.

Next, we examined the presence of coagulation factors in lung fluids. Among them, fibrinogen, prothrombin, antithrombin-III, factor XII, and plasminogen are routinely present in all BALF samples; other coagulation factors are less abundant and mostly undetectable by mass spectrometry (Table S1). Importantly, similar levels of coagulation factors are found in the vaccinated and healthy donors; both are less abundant than those in the non-vaccinated individuals (Fig. 2C). Furthermore, the abundance of common plasma proteins is also less in the vaccinated than in the non-vaccinated BALF (Fig. S2), consistent with the reduction of plasma infiltration in vaccinated COVID lungs. Further analysis of the mass spectrometry data from the longitudinal COVID-19 studies by Kanth et al. also supports the presence of lower levels of BALF coagulation factors in the vaccinated than non-vaccinated individuals. Specifically, both fibrinogen and prothrombin were significantly less abundant in the vaccinated and healthy group than in the non-vaccinated group (Fig. 3A and B). In addition, the level of fibrinogen-α, -β, and -γ in both vaccinated and non-vaccinated BALF decreased as individuals progressed from the acute to recovery phase (Fig. 3C).

**Fig 4:**
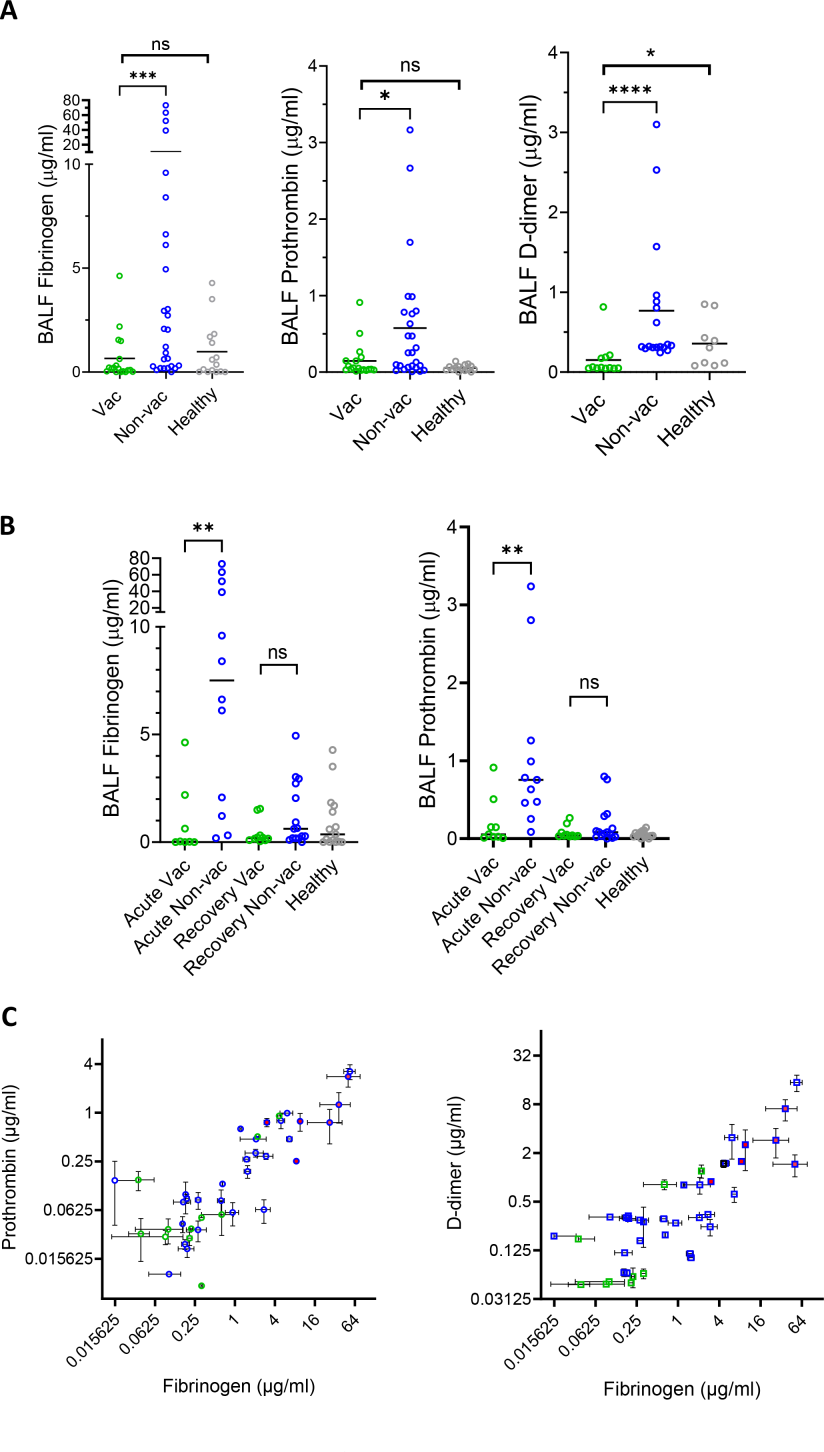
Concentrations of fibrinogen, prothrombin, and D-dimer as determined by ELISA. (**A**) Fibrinogen, prothrombin, and D-dimer concentrations in vaccinated (green circle), non-vaccinated (blue circle), and healthy (gray circle) BALF samples. (**B**) concentrations of BALF fibrinogen and prothrombin as in panel A except that the samples were further divided into acute (in the first 3 weeks of symptom onset) and recovery (after 3 weeks of symptom onset) groups. Statistics were calculated using a two-tailed non-parametric Mann-Whitney test. When the differences between vac and non-vac are analyzed by two-way ANOVA with the fibrinogen, prothrombin, and D-dimer panels combined, it results in a *P* value of **** between vaccinated and non-vaccinated groups. (**C**) Correlated distribution in BALF concentrations of fibrinogen versus prothrombin, fibrinogen versus D-dimer from BALF samples. Vaccinated and non-vaccinated BALF are shown in green and blue symbols, respectively. The BALF exhibited fibrin clotting under infection and is labeled red. Simple linear regressions on prothrombin and D-dimer versus fibrinogen resulted in R squared values of 0.8254 and 0.5833, respectively, with both *P* values for fitted slopes <0.0001. *P < 0.05, **P < 0.01, ***P < 0.001, ****P < 0.0001; ns, not significant.

To further validate the proteomic analyses on fibrinogen and prothrombin levels, we measured fibrinogen, prothrombin, and D-dimer concentration from the vaccinated, non-vaccinated COVID, as well as healthy BALF samples by ELISA (Fig. S4). Unlike their plasma concentrations, which varied less than threefold among individuals, the BALF fibrinogen and prothrombin concentrations varied up to 500-fold (Fig. 4A; Fig. S4). Overall, most fibrinogen concentrations varied from less than 50 to 500 ng/mL in the vaccinated, but to greater than 50 µg/mL in non-vaccinated COVID BALF samples. Likewise, most prothrombin concentrations ranged from 10 to 200 ng/mL in vaccinated BALF but to ~3 µg/mL in non-vaccinated samples (Fig. 4A; Fig. S4). Consistent with the mass spectrometry data, concentrations from vaccinated individuals are similar to those from healthy donors but are significantly lower than those from non-vaccinated individuals (Fig. 4A). When the vaccinated and non-vaccinated samples are further separated into the acute and recovery phases, it shows that vaccination primarily reduced the acute phase coagulation factor concentrations (Fig. 4B). Despite their wide variation in concentrations, both prothrombin and D-dimer levels are correlated with those of fibrinogen (Fig. 4C).

### Viral-induced fibrin was absent in vaccinated BALF and correlated with COVID severity

**Fig 5:**
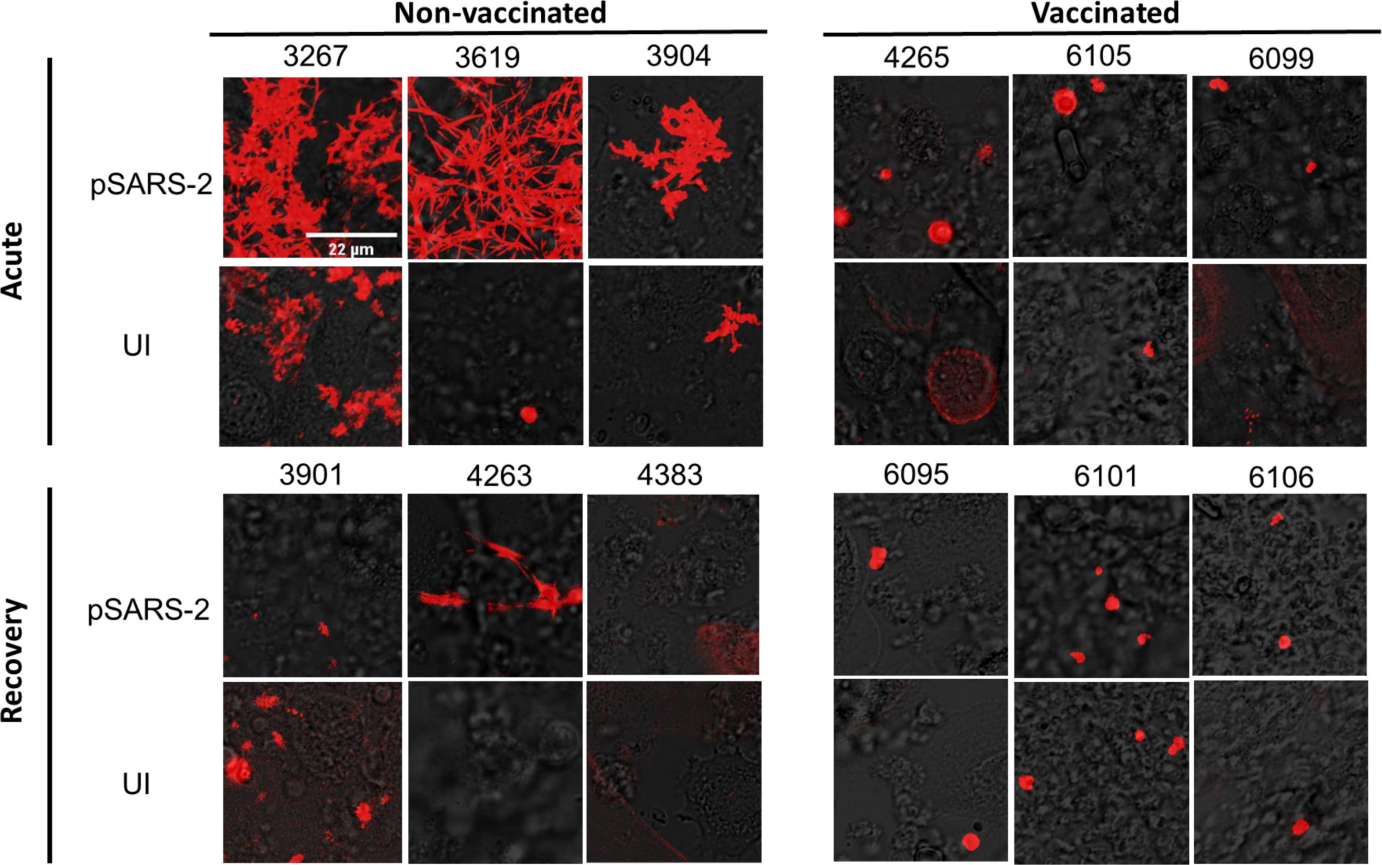
SARS-CoV-2 infection-induced fibrin deposition in BALF samples. HSAEC cells were infected with pseudo-typed Omicron SARS-CoV-2 viruses (SARS-2) or mock (UI) for 24–48 h and replaced media with various BALF in the presence of clotting buffer for ~4 h before taking confocal images.

**Fig 6:**
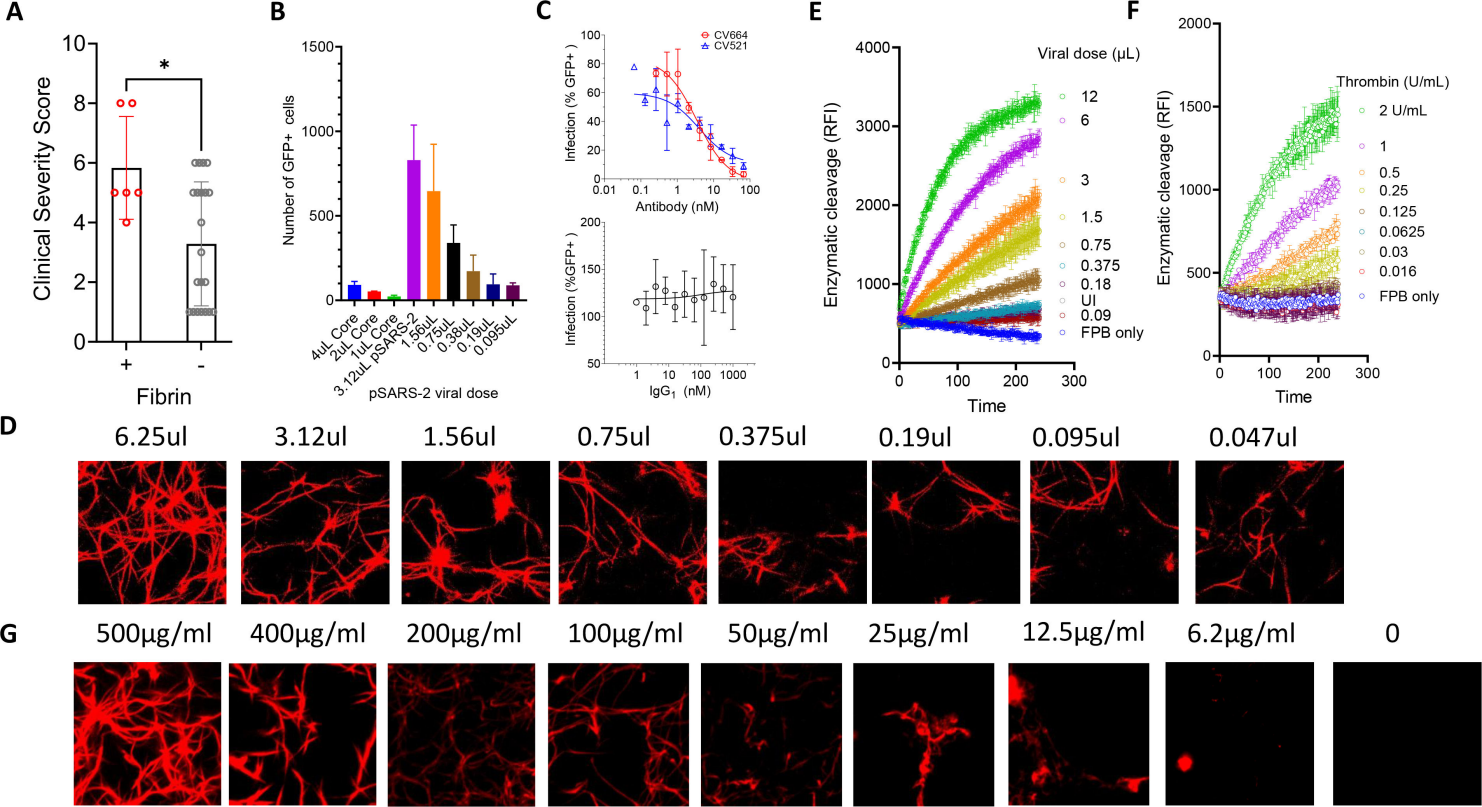
Titration of SARS-CoV-2 infection and fibrin clotting. (**A**) Association of COVID clinical score with the viral-induced fibrin in BALF. (**B–C**) Titration of pSARS-2 viral dose in infection of ACE2-293T cells (**B**) and in the presence of serially diluted neutralization antibody CV664, CV521, or IgG1 (**C**). The inhibition constant (IC50) of SARS-CoV-2 neutralizing antibodies CV664 and CV52 is 3.5 and 4.0 nM, respectively. (**D**) Confocal images visualize the fibrin clot formation from titration doses of pSARS-2 infected HSAEC cells. (**E–F**) Enzymatic cleavage of fluorogenic fibrinogen-β peptide by supernatants from titration dose of pSARS-2 infected or uninfected (UI) HSAEC cells (**E**), or thrombin (**F**). The cleavage was indicated by time-dependent relative fluorescence intensity. (**G**) Formation of the viral-induced fibrins with varying fibrinogen concentrations. All conditions were infected identically with omicron pSARS-2 virus but with different amounts of fibrinogen for fibrin clotting. *P < 0.05

Elevated fibrinogen and prothrombin levels can induce fibrin formation in the presence of SARS-CoV-2 infection of primary airway epithelial cells. This viral-induced fibrin depends on the activation of prothrombin by TMPRSS family proteases on infected airway epithelial cells (40). Both ELISA and proteomic data showed reduced levels of fibrinogen and prothrombin in vaccinated compared to non-vaccinated BALF, suggesting less risk of fibrin deposition in vaccinated BALF. To address this, we evaluated the tendency of vaccinated and non-vaccinated BALF to develop fibrin clots in the presence of SARS-CoV-2 infections. Normal human bronchial/tracheal epithelial (NHBE) or small airway epithelial (HSAEC) cells were infected with SARS-CoV-2 pseudovirus at a dose of 2–5 copies viral RNA/cell in 384-well cell culture plates for 24 h before replacing the cell culture media with 1:1 mix of 20-fold concentrated BALF and clotting buffer (20 mM HEPES, 137 mM NaCl, 5 mM CaCl_2_) for 2–4 h at 26°C to observe fibrin clotting. The samples were then fixed with 2% paraformaldehyde, and fibrin clots were imaged using a confocal microscope. Fibrin clots were observed in 3 out of 11 non-vaccinated acute BALF in the presence of the viral infections (Fig. 5; Fig. S5). In contrast, no fibrin clots were observed in any of the vaccinated BALF (Fig. 5; Fig. S5), suggesting that the concentrations of fibrinogen and prothrombin in the vaccinated BALF were insufficient to support fibrin clot formation. These results indicate that the COVID vaccine protects against SARS-CoV-2 infection-induced pulmonary fibrin deposition. It is worth noting that the majority of non-vaccinated BALF with a lower concentration of fibrinogen did not form the viral-induced fibrin. Fibrin formations were observed only in non-vaccinated BALF containing the highest levels of coagulation factors (Fig. 4C). When the association between the presence of viral-induced fibrin and COVID clinical severity is analyzed regardless of the vaccination status, the formation of fibrin was found to correlate with COVID severity (Fig. 6A), suggesting pulmonary fibrin deposition contributes to severe COVID disease.

### Protective mechanism of vaccination against viral-induced fibrin deposition

The association between fibrin deposition and COVID severity prompted us to examine the dependence of fibrin clotting on the viral doses. We infected ACE2-293T with either titration doses of pseudotyped SARS-CoV-2 (pSARS-2) or a constant viral dose in the presence of titration amounts of neutralizing antibodies (CV664 and CV521) (51). As expected, the infection of ACE2-293T cells correlated with the viral dose and neutralization antibodies CV664 and CV521 but not IgG_1_ inhibited the viral infections with IC_50_ of 3.4 and 4.0 nM, respectively (Fig. 6B and C; Fig. S6A through C). Likewise, the extent of fibrin clotting was also dependent on the viral dose (Fig. 6D; Fig. S7A and B). Additionally, when the supernatants from the titration dose pSARS-2 infected HSAEC cells were used to cleave a fluorogenic fibrinogen-β peptide, corresponding to the thrombin cleavage region, it resulted in a viral-dose-dependent cleavage like the peptide cleavage by thrombin (Fig. 6E and F), suggesting that the prothrombin activation depends on the viral dose. Thus, while vaccination-induced neutralization antibodies may be insufficient to prevent infections (52, 53), vaccination leads to partial neutralization of SARS-CoV-2 viral titers, resulting in diminished prothrombin activation and reduced infiltration of fibrinogen compared to non-vaccinated naïve infections.

### Pulmonary fibrinogen level indicates the risk of developing viral-induced fibrin

Unlike their plasma concentrations that varied less than threefold in SARS-CoV-2 infections, pulmonary fibrinogen concentrations varied more than 500-fold from less than 20 ng/mL to greater than 10 µg/mL in non-vaccinated COVID BALF (Fig. S4). Since fibrinogen, prothrombin, and D-dimer concentrations in BALF are correlated and fibrin clotting was observed in samples with highest concentrations of all three (Fig. 4C), we then asked if pulmonary rather than plasma fibrinogen level serves a better indication for the risk of fibrin deposition in SARS-CoV-2 infected lung and if a threshold level of fibrinogen exists above which fibrin clotting becomes significant. To address the minimum concentration of fibrinogen required to support viral-induced fibrin deposition, we assessed the extent of SARS-CoV-2-induced fibrin clot formation with titration concentrations of fibrinogen between 10 and 500 µg/mL, corresponding to approximately 0.5–25 µg/mL fibrinogen in BALF in the presence of a constant dose of a GFP-expressing omicron-spike pSARS-2 virus. Indeed, the amount of viral-induced fibrin clot formation was proportional to the fibrinogen concentration (Fig. 6G), and the viral-induced fibrin formation occurred at above ~50 µg/mL of fibrinogen (or 2–5 µg/mL of fibrinogen in BALF), below which no significant fibrin was observed (Fig. 6G; Fig. S4A and S7C). This suggests a protective mechanism of vaccination against severe COVID is through reducing pulmonary fibrinogen and prothrombin into infected lungs. Indeed, the fibrinogen concentrations in vaccinated BALF are between 50 and 5 µg/mL (Fig. 4A), generally below the threshold of fibrin clotting. Among the non-vaccinated samples, the majority of their fibrinogen concentrations are also below the fibrin clotting threshold, with only the highest ones exceeding the clotting threshold and supporting viral-induced fibrin clotting, suggesting these individuals are at risk of developing pulmonary fibrin deposition. Taken together, we suggest that pulmonary fibrinogen concentration serves as a biomarker for the risk of developing COVID-associated lung fibrin deposition.

## DISCUSSION

As COVID-19 evolves from pandemic to recurring seasonal infections, vaccines and antiviral drugs are the primary health care measures to counter SARS-CoV-2 infections. Despite the frequent occurrence of breakthrough infections, vaccines protected against severe COVID diseases. Understanding the mechanism of this protection is key to developing therapeutic treatment against severe COVID. Early publications suggested COVID vaccination reduced the overt immune responses associated with SARS-CoV-2 infections (11–13), thus lessened pulmonary immunopathology. However, vaccination also benefited the immunocompromised population (21, 22). Further complicating our understanding is the lack of a reliable biomarker for severe COVID-associated hypercoagulation.

**Fig 7:**
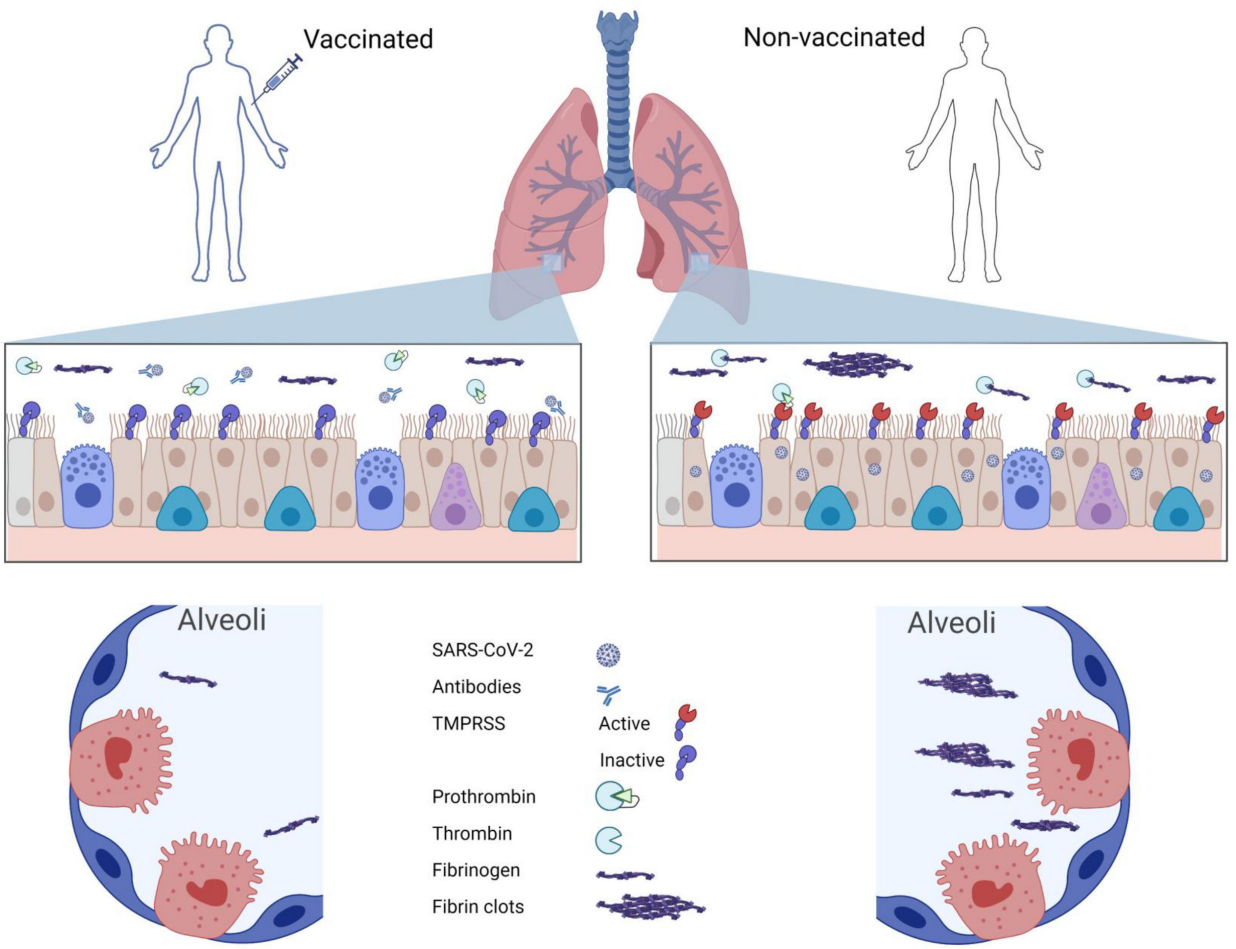
Schematic showing the protective effect of vaccination in preventing fibrin deposition associated with SARS-CoV-2 infection. Vaccine-induced neutralizing antibodies partially neutralize infectious viral titer, resulting in lower cellular activation of TMRPSS proteases from infected cells, leading to lower activation of prothrombin. In addition, reduced inflammation reduces plasma infiltration of fibrinogen and prothrombin to the level insufficient to trigger fibrin clotting.

Here, we investigated the protective mechanisms of SARS-CoV-2 vaccines against severe COVID-19 diseases. Early autopsy data showed the presence of fibrosis in diseased lungs (25–27). However, clinical use of anticoagulant, including low molecular weight heparin, failed to mitigate severe COVID-associated mortality (16). We introduced here a model for severe COVID-associated pulmonary hypercoagulation based on SARS-CoV-2 infection-induced fibrin formation and showed it correlated with disease severity (Fig. 6A) (40). Using this model, we investigated the influence of vaccination on the viral-induced fibrin deposition. We found that plasma coagulation indices, including PT, PTT, as well as plasma concentrations of fibrinogen and prothrombin remained similar between COVID and healthy, or vaccinated and non-vaccinated COVID groups. In contrast, vaccination reduced pulmonary inflammation and plasma infiltrations, resulting in lower pulmonary fibrinogen, prothrombin, and D-dimer concentrations in vaccinated than non-vaccinated COVID BALF. Importantly, vaccinations protected against the viral-induced fibrin formation, suggesting a protective mechanism of SARS-CoV-2 vaccine by reducing the risk of pulmonary fibrin deposition. The benefit of the vaccine appears twofold. First, the vaccine reduces the viral load and, consequently, less viral-induced prothrombin activation. Second, the vaccine reduces infiltration of coagulation components into infected lungs (Fig. 7).

SARS-CoV-2 infection leads to pneumonia and DAD with characteristic fibrin-rich hyaline membranes. Our finding supports that the viral-induced fibrin deposition contributed to the formation of pulmonary hyaline membranes. The formation of viral-induced fibrin deposition depends on both viral load and inflammation. Higher viral loads would lead to increased activation of TMPRSS proteases in infected lung epithelial cells, as well as increased inflammatory infiltration of fibrinogen and prothrombin into infected alveolar space, leading to the activation of prothrombin and fibrin deposition. The presence of vaccine-induced neutralization antibodies can minimize viral-induced fibrin formation through reducing both viral activation of TMPRSS proteases and inflammatory infiltration of coagulation components. As the levels of BALF fibrinogen and prothrombin correlated with viral-induced fibrin formation, we speculate that their pulmonary but not plasma concentrations serve as a better marker for clinical risk of severe COVID. We estimated the threshold fibrinogen concentration to be ~50 µg/mL for developing viral-induced pulmonary fibrin clots. Consistently, healthy donors have less than 10 µg/mL fibrinogen in their lung, well below the clotting threshold. The majority of the non-vaccinated lavages do not contain high levels of fibrinogen to result in fibrin deposition. Some non-vaccinated lavages show similar levels of fibrinogen and prothrombin as vaccinated or healthy samples, suggesting the presence of strong natural immunity is sufficient to prevent fibrin deposition and DAD. However, a small number of non-vaccinated lavages, ~10% in this small study, exhibited viral-induced fibrin formation.

In summary, the investigation of pulmonary coagulation factors and their involvement in viral-induced fibrin clot formation in vaccinated and non-vaccinated BALF revealed a vaccine-mediated protective mechanism against SARS-CoV-2 induced pulmonary fibrin deposition and suggests the use of pulmonary fibrinogen concentration as a biomarker for severe COVID.

## MATERIALS AND METHODS

### Clinical samples

BAL and plasma were collected from 43 COVID patients who tested positive with SARS-CoV-2 infections by PCR under IRB-approved protocols. Thirty of the 43 COVID BAL samples were collected as part of COVID ARC-19 (Cardiopulmonary Inflammation and Multi-System Imaging During the Clinical Course of COVID-19 Infection in Asymptomatic and Symptomatic Persons, NCT04401449) studies from the National Institutes of Health. The details of COVID-ARC-19 patients and their responses to SARS-CoV-2 infection were described previously (46). Thirteen of the COVID BAL samples were collected through a CLIA-approved clinical BAL laboratory at Indiana University under protocol 1011003397R010. Eighteen of the COVID patients received either mRNA-1273 (Moderna), BNT162b2 (Pfizer), or Ad26.COV2.S (Johnson & Johnson) vaccinations and thus were regarded as having breakthrough infections, while 25 of the COVID patients did not receive prior vaccination before the study. Plasma samples were collected from a subgroup of the COVID-ARC-19 patients. Eighteen of the BAL samples collected within the first 20 days of COVID symptom onset are designated as acute, and 19 of the BAL samples collected between 3 and 8 weeks are designated as recovery samples, and 6 samples collected after 8 weeks are designated as convalescent samples. Most of the samples were collected during the early pandemic between 2020 and 2021, before omicron infections. BAL and plasma of healthy controls were collected from separate studies or acquired from Audubon Biosciences (New Orleans, LA). BALF was obtained by centrifugation to remove cells and other insoluble debris in BAL samples. All samples were collected with patient consent and used without individual identifications. The clinical scores of COVID severity were assigned 1 through 8 corresponding to 1–2: non-hospitalized mild symptom for no oxygen needed and home oxygen use cases; 3–5: hospitalized moderate disease for no oxygen, no oxygen but ongoing care, and oxygen by mask or nasal prongs; 6–7: hospitalized severe disease for high flow non-invasive oxygen, mechanical ventilation; 8: death after hospitalization. Blood coagulation profiles for all clinical samples, including PT, activated PTT, INR, as well as concentrations of CRP, D-dimer, and fibrinogen, were measured within ~4 weeks of symptom onset using an automated hemostasis platform (ACLTOP750 CTS, Werfen) in the Department of Laboratory Medicine at the NIH Clinical Center. Other parameters on the clinical samples, including fibrinogen, prothrombin, additional D-dimer concentrations, and SARS-CoV-2 spike-specific IgG, were measured by ELISA in-house as described below.

### Reagent

ACE2-expressing HEK 293T cells (referred to as ACE2-293T) were purchased from Genecopoeia, Inc., MD. Normal human bronchoalveolar epithelial (NHBE, catalog PCS-300-010) cells, human small airway epithelial cells (HSAEC, catalog PCS-301-010) and their culture media components were purchased from American Type Culture Collection (ATCC, https://www.atcc.org).

### Production of SARS-CoV-2 pseudoviruses

For the production of pSARS-2, HEK 293T cells were cultured at a density of 2.5 × 10^6^ cells in 10 cm plates in Dulbecco’s Modified Eagle’s Medium supplemented with 10% heat-inactivated fetal bovine serum, 2 mM L-glutamine, and 1% penicillin-streptomycin in a 37°C incubator with 5% CO_2_. Upon near confluence, cells were co-transfected with a SARS-CoV-2 spike protein plasmid and an env-/nef- GFP-expressing HIV NL4-3 core plasmid, in which the viral nef gene is replaced with an EGFP coding sequence, using Lipofectamine 3000 according to the manufacturer’s protocol (54, 55). Plasmids encoding SARS-CoV-2 spike genes, including Wuhan (56) and omicron (B.1.1.529) BA.2 strains, were obtained from Addgene (https://www.addgene.org). Supernatants containing pseudovirus particles were harvested 48 h post-transfection and concentrated 100-fold using the PEG Virus precipitation kit (MAK343-1KT, Sigma-Aldrich Co., MO). The titer of SARS-CoV-2 pseudovirus was estimated by reverse transcription-PCR in numbers of RNA copies/mL. In brief, RNA was extracted from 50 uL concentrated pseudovirus using the Qiagen RNeasy Mini Kit, and cDNA was generated using a C1000 Touch Thermal Cycler (BIO-RAD, CA 94547) with ABI High-Capacity cDNA Reverse Transcription Kit following the manufacturer’s protocol. HIV-1 NL4-3 LTR was amplified using TaqMan HIV-1 LTR primer/probe sets (Pa03453409_s1) from ThermoFisher with 50 ng cDNA as template. Samples were run in duplicate using a QuantStudio 6 Pro Real-Time PCR System (ThermoFisher, MA 02451) together with a serial dilution of a known copy number HIV DNA as standards. The pseudovirus titers were between 10^8^ and 10^9^ copies of RNA/mL. The use of SARS-CoV-2 viruses was approved by the NIH inter-institute Biosafety Committee (IBC) under protocol RD-20-VI-12.

### Infection of ACE2-293T cells with SARS-CoV-2 viruses

ACE2-293T cells were grown either in 384-well or 96-well cell culture plates at appropriate densities to near confluence. For SARS-CoV-2 pseudovirus infections, cells were infected with a titration dose of pSARS-2 between 1 and 100 copies of viral RNA/cell in fresh culture media for 24–72 h, and the infections were measured by the number of GFP+ cells. For antibody neutralization of infections, near-confluent ACE2-293T cells in a 384-well plate were infected with ~10–40 copies of viral RNA/cell in their growth media in the presence of titration amounts of antibodies between 0.2 and 70 nM or IgG between 1 and 1,000 nM.

### Fibrin clotting turbidity assay

Purified fibrinogen from human plasma (Sigma-Aldrich, MO) was dissolved in 100 mM NaCl, 20 mM HEPES buffer. The solution was incubated at 37°C for 10 min, then filtered through a 0.45 um syringe filter. The solution was stored at 4°C for 30 min, then filtered again to remove aggregates. Concentration was measured using nanodrop, then the solution was aliquoted and frozen at −20°C. Clot formation was assayed using fibrinogen solution diluted to 1.5 µM concentration in clotting buffer (20 mM HEPES, 137 mM NaCl, 5 mM CaCl_2_). Diluted fibrinogen was added to thrombin enzyme (5 U/mL, Sigma) (positive control) or infected/uninfected HSAEC cells and placed in a plate reader. The absorbance was measured at 350 nm wavelength continuously with 2 min intervals for 4–10 h with Synergy_H1 (BioTek) plate reader. Fibrin clot formation causes scattering of light that passes through the solution, which increases the turbidity. For infection-induced fibrin clotting assays, primary NHBE or HSAEC cells were grown in 384-wells at 1,000 cells/well or in a 96-well plate at 4,000 cells/well in their culture media, consisting of airway epithelial cell basal medium supplemented with bronchial epithelial growth kit as recommended by ATCC, until 60%–80% confluence. The cells were infected with various strains (Wuhan or omicron BA.2) of pSARS-2 at ~1–20 copies of RNA per cell for 24 h before replacing growth media with either fibrinogen or concentrated BALF in clotting buffer. To use BALF samples in the fibrin clotting assay, BALF samples were first concentrated 20-fold using 3 K molecular weight cutoff Amicon Ultra-4 concentration filters (Millipore catalog UFC800396) at 4°C to approximate the content in lung epithelial lining fluid. The growth media from the infected cells were replaced with a mixture of half volume of concentrated BALF and half volume of the clotting buffer. For confocal imaging, fibrin clotting assays were monitored by absorption at 350 nm wavelength for 2–4 h and then fixed with 2% paraformaldehyde before imaging by confocal microscope.

### Enzymatic cleavage of a fluorogenic fibrinogen-β peptide

The fluorogenic fibrinogen-β peptide, FPB, was synthesized by Biomatik as dabcyl-SARGHRPLE-edans, corresponding to amino acids 42–49 of human fibrinogen-β encompassing the thrombin cleavage site. The cleavage of FPB peptide was carried out by mixing 10 µM of the peptide with 20 µL of infected or uninfected HSAEC supernatants and 5 µL of the assay buffer containing 50 mM Tris, 0.01% Tween 20 at pH 9. The enzymatic cleavage reactions were monitored using a Synergy_h1 fluorescent plate reader (BioTek) with 340 nm excitation and 490 nm emission wavelengths for 4 h at 37°C.

### Proteomics analyses of BALF by mass spectrometry

Fifteen microliter aliquots of BALF samples were mixed with 5 µL 4× LDS-sample buffer and applied onto a 4%–12% Nupage gel with MOPs running buffer. The run stopped after the samples migrated approximately ¼ distance into the gel. Each lane of the gel was sliced into smaller pieces and subjected to destaining, reducing/alkylation, and in-gel trypsin digestion. The extracted peptides were applied for liquid chromatography-tandem mass spectrometry analysis using either a Thermo Orbitrap Fusion or a Thermo Orbitrap Fusion Lumos operated with an in-line Thermo nLC 1200 and an EASY-Spray ion source. Peptides were separated using a 2 cm Pepmap 100 C18 trap column and a 25 cm Easy-spray Pepmap 100 C18 analytical column. MS/MS data acquisitions were operated at a 120,000 resolution (m/z 200) with a scan range of 350–1,950 m/z and CID fragmentation. All data were processed using Proteome Discoverer v2.4 (Thermo Scientific) with a SEQUEST HT search against the Uniprot KB/Swiss-Prot Human Proteome (02/2021) and common contaminants (theGPM.org) using a 5 ppm precursor mass tolerance and a 0.5 Da fragment tolerance. Dynamic modifications included in the search were limited to oxidation [M], deamidation [NQ], and acetylation [Protein N-terminal] while carbamidomethylation [C] was the only static modification utilized. Peptides and proteins were filtered at a 1% false discovery rate using a target-decoy approach with a two-peptide per protein minimum. Relative protein abundance was estimated from an average of its top three unique peptide intensities as determined by chromatographic area-under-the-curve and normalized by total intensity of all peptides. The sum of abundances in a data set is normalized to 1,000,000. The differential abundance is calculated as a percentage of difference in abundance: by dividing the difference in abundance between a protein in one sample and the average abundance of the protein by the average abundance of the protein from all healthy samples. The list of proteins used for the differential abundance heatmap analysis includes the ones with average healthy abundance greater than 25 and all non-zero abundance in the acute COVID sample. The heatmaps display the fold change in abundance relative to the average of each protein. Figure 3 is generated from analysis of fibrinogen and prothrombin in the quantitative proteomics data collected on acute, recovery, and convalescent BALF samples using data-independent acquisition mass spectrometry (DIA-MS) by Kanth et al. (46). The sum of DIA-MS abundances from each individual sample was similarly normalized to 1,000,000.

### Measuring fibrinogen, prothrombin, D-dimer, and IgG concentrations by ELISA

ELISA assays were used to determine the levels of fibrinogen (Abcam, ab108841), total IgG (Abcam, ab195215), prothrombin (Innovative Research, IHUFIIKTT), SARS-CoV-2 Spike IgG (Invitrogen, BMS2325), and D-dimer (Abcam, ab260076) present in human BALF and serum samples. The samples were diluted with kit-specific assay diluents. BALF sample dilutions ranged from D-Dimer (1:10 and 1:100), prothrombin, SARS-CoV-2 Spike IgG, and fibrinogen (1:50 and 1:500), and total IgG (1:1,000 and 1:10,000). Serum sample dilutions were ten times more dilute for all conditions. The assays were carried out following the manufacturer’s protocols.

### SARS-CoV-2 neutralization antibody titer assay

SARS-CoV-2 neutralizing antibody titers were measured by a competitive ELISA assay based on the blocking of biotinylated ACE2 binding to immobilized SARS-CoV-2 RBD by serum neutralizing antibodies. The ELISA was performed and analyzed according to the manufacturer’s instructions (Thermo Fisher Scientific, catalog BMS2326). In brief, samples were run undiluted (BAL-F) or diluted 1:50 (plasma). Positive inhibition is defined as greater than or equal to 20%, while negative results are defined as less than 20%. Inhibition calculations are based on the change in absorbance relative to the negative control wells.

### Imaging of fibrin fibers by confocal microscopy

Fibrinogen was labeled with a fluorescent dye TAMRA-SE (Thermo Fisher Scientific, catalog c1171) according to the manufacturer’s protocol. Trace of fluorescent TAMRA-fibrinogen was added to fibrin clotting assays at 80 µg/mL concentration or mixed with unlabeled fibrinogen at a 1:6 ratio. Images were taken on a Zeiss LSM 880 confocal microscope equipped with Plan-Apochromat 20×/0.8 M27 objective. Z-stacks were performed to image fibrin formation. After acquisition, maximum intensity projections of the z-stacks were made using Fiji.

## Data Availability

The proteomic data sets reported in Table S1 are available at PRIDE (Proteomics Identifications Database, https://www.ebi.ac.uk/pride/) via ProteomeXchange under submission PXD066557.

## SUPPLEMENTAL MATERIAL

Supplemental figures jvi.00633-25-s0001.pdf Figures S1 to S7.

Table S1 jvi.00633-25-s0002.xlsx Proteomics analysis of COVID BALF samples.

ASM does not own the copyrights to Supplemental Material that may be linked to, or accessed through, an article. The authors have granted ASM a non-exclusive, world-wide license to publish the Supplemental Material files. Please contact the corresponding author directly for reuse.
