# Supplementary material for: Vaccination protects against COVID-associated pulmonary fibrin deposition
Source: J Virol. 2025 Nov 6;99(11):e00633-25. doi: 10.1128/jvi.00633-25 (PMC12645957; doi:10.1128/jvi.00633-25)
Supplement: Supplemental figures — Figures S1 to S7. [file jvi.00633-25-s0001.pdf]

Supplemental Figures

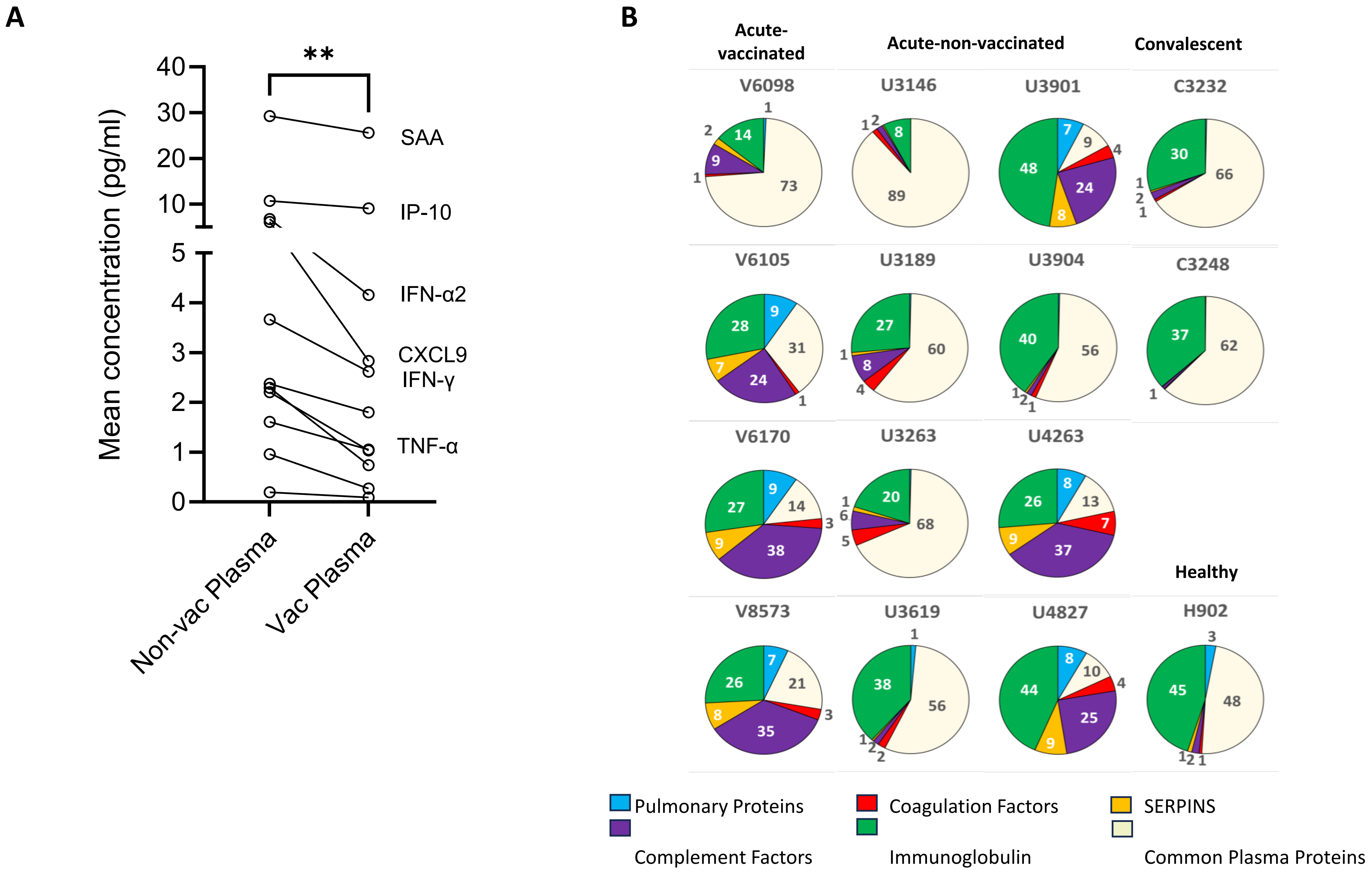
A
B
Mean concentration (pg/ml)
**
SAA
IP-10
IFN-α2
CXCL9
IFN-γ
TNF-α
Non-vac Plasma
Vac Plasma
Acute-vaccinated
Acute-non-vaccinated
Convalescent
Healthy
V6098
U3146
U3901
C3232
V6105
U3189
U3904
C3248
V6170
U3263
U4263
V8573
U3619
U4827
H902
Pulmonary Proteins
Complement Factors
Coagulation Factors
Immunoglobulin
SERPINS
Common Plasma Proteins

Supplemental Figure 1. Cytokine concentrations in COVID plasma and proteomic analysis of COVID BALF. A) Cytokine concentrations in non-vaccinated and vaccinated COVID-ARC-19 plasma samples as determined using multiplex cytokine bead array analysis by flow cytometry. The vaccinated and non-vaccinated plasma samples exhibited cluster 1 and cluster 2 inflammatory profiles, respectively in Kanth et al. The cytokines include (from top to bottom) SAA, IP-10, IFN-α2 (fg/ml),CXCL9,IFN-γ, IL-8,IL-23, IL-6,TNF-α, IL-10, and IL-1β. B) Proteomic analyses of acute (vaccinated, non-vaccinated), convalescent and healthy BALF. Pie charts show relative abundance of different category of plasma proteins found in acute (vaccinated, non-vaccinated) and convalescent COVID, as well as healthy BALF.

Supplemental Figure 2

A

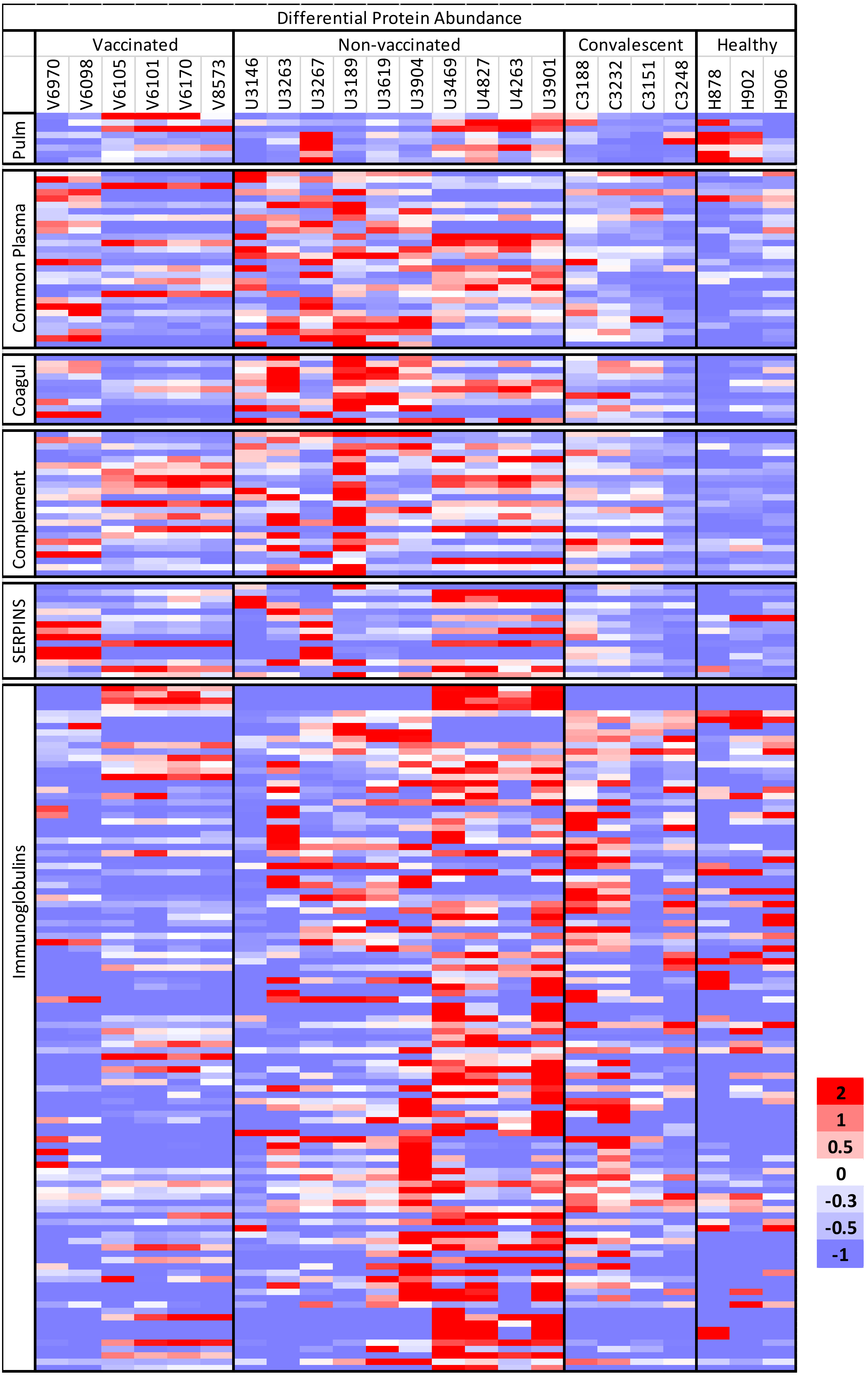

B

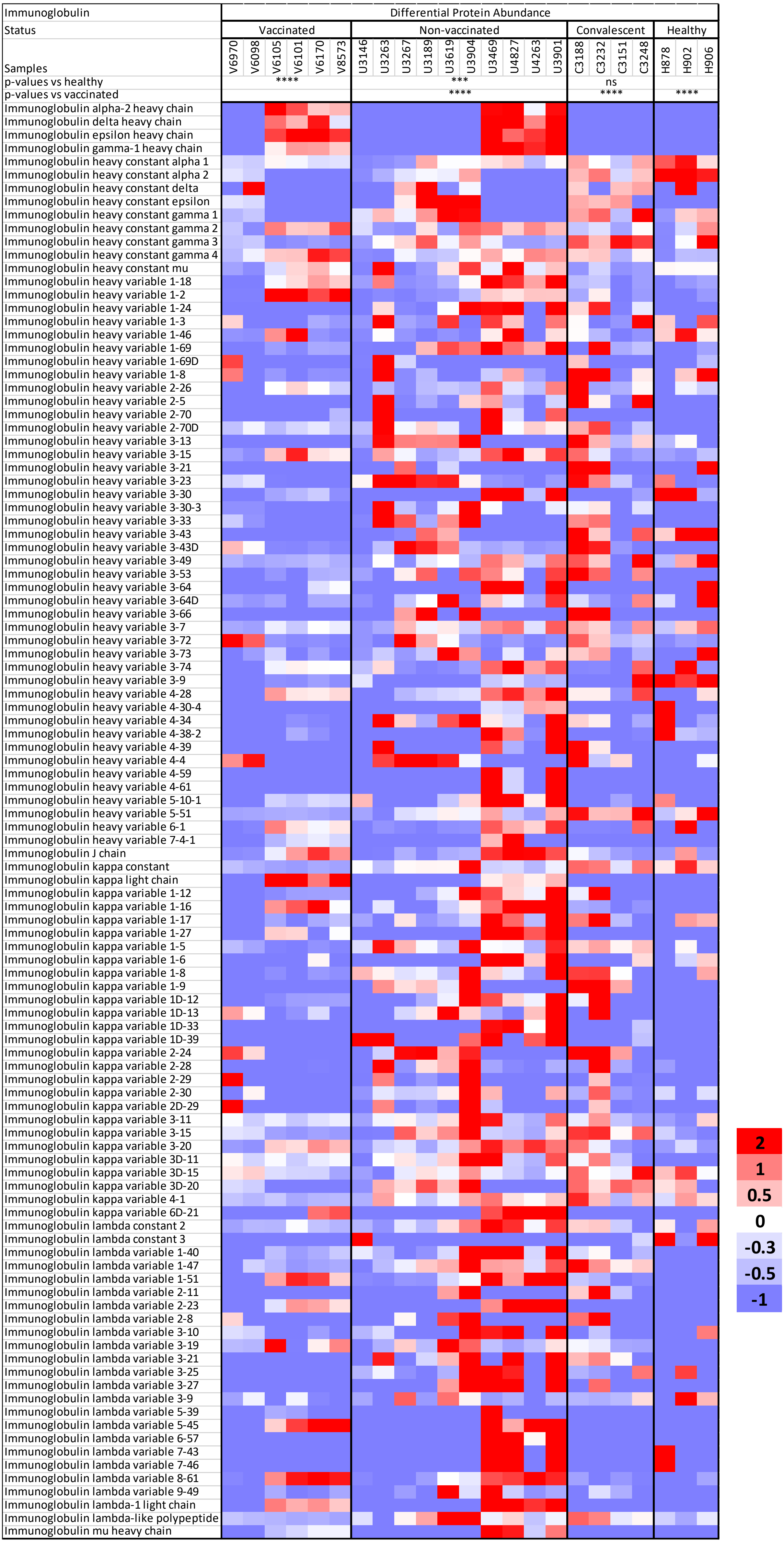
Immunoglobulin
Differential Protein Abundance
Status
Vaccinated
Non-vaccinated
Convalescent
Healthy
Samples
V6970
V6098
V6105
V6101
V6170
V8573
U3146
U3263
U3267
U3189
U3619
U3904
U3469
U4827
U4263
U3901
C3188
C3232
C3151
C3248
H878
H902
H906
p-values vs healthy
****
***
ns
p-values vs vaccinated
****
****
****
Immunoglobulin alpha-2 heavy chain
Immunoglobulin delta heavy chain
Immunoglobulin epsilon heavy chain
Immunoglobulin gamma-1 heavy chain
Immunoglobulin heavy constant alpha 1
Immunoglobulin heavy constant alpha 2
Immunoglobulin heavy constant delta
Immunoglobulin heavy constant epsilon
Immunoglobulin heavy constant gamma 1
Immunoglobulin heavy constant gamma 2
Immunoglobulin heavy constant gamma 3
Immunoglobulin heavy constant gamma 4
Immunoglobulin heavy constant mu
Immunoglobulin heavy variable 1-18
Immunoglobulin heavy variable 1-2
Immunoglobulin heavy variable 1-24
Immunoglobulin heavy variable 1-3
Immunoglobulin heavy variable 1-46
Immunoglobulin heavy variable 1-69
Immunoglobulin heavy variable 1-69D
Immunoglobulin heavy variable 1-8
Immunoglobulin heavy variable 2-26
Immunoglobulin heavy variable 2-5
Immunoglobulin heavy variable 2-70
Immunoglobulin heavy variable 2-70D
Immunoglobulin heavy variable 3-13
Immunoglobulin heavy variable 3-15
Immunoglobulin heavy variable 3-21
Immunoglobulin heavy variable 3-23
Immunoglobulin heavy variable 3-30
Immunoglobulin heavy variable 3-30-3
Immunoglobulin heavy variable 3-33
Immunoglobulin heavy variable 3-43
Immunoglobulin heavy variable 3-43D
Immunoglobulin heavy variable 3-49
Immunoglobulin heavy variable 3-53
Immunoglobulin heavy variable 3-64
Immunoglobulin heavy variable 3-64D
Immunoglobulin heavy variable 3-66
Immunoglobulin heavy variable 3-7
Immunoglobulin heavy variable 3-72
Immunoglobulin heavy variable 3-73
Immunoglobulin heavy variable 3-74
Immunoglobulin heavy variable 3-9
Immunoglobulin heavy variable 4-28
Immunoglobulin heavy variable 4-30-4
Immunoglobulin heavy variable 4-34
Immunoglobulin heavy variable 4-38-2
Immunoglobulin heavy variable 4-39
Immunoglobulin heavy variable 4-4
Immunoglobulin heavy variable 4-59
Immunoglobulin heavy variable 4-61
Immunoglobulin heavy variable 5-10-1
Immunoglobulin heavy variable 5-51
Immunoglobulin heavy variable 6-1
Immunoglobulin heavy variable 7-4-1
Immunoglobulin J chain
Immunoglobulin kappa constant
Immunoglobulin kappa light chain
Immunoglobulin kappa variable 1-12
Immunoglobulin kappa variable 1-16
Immunoglobulin kappa variable 1-17
Immunoglobulin kappa variable 1-27
Immunoglobulin kappa variable 1-5
Immunoglobulin kappa variable 1-6
Immunoglobulin kappa variable 1-8
Immunoglobulin kappa variable 1-9
Immunoglobulin kappa variable 1D-12
Immunoglobulin kappa variable 1D-13
Immunoglobulin kappa variable 1D-33
Immunoglobulin kappa variable 1D-39
Immunoglobulin kappa variable 2-24
Immunoglobulin kappa variable 2-28
Immunoglobulin kappa variable 2-29
Immunoglobulin kappa variable 2-30
Immunoglobulin kappa variable 2D-29
Immunoglobulin kappa variable 3-11
Immunoglobulin kappa variable 3-15
Immunoglobulin kappa variable 3-20
Immunoglobulin kappa variable 3D-11
Immunoglobulin kappa variable 3D-15
Immunoglobulin kappa variable 3D-20
Immunoglobulin kappa variable 4-1
Immunoglobulin kappa variable 6D-21
Immunoglobulin lambda constant 2
Immunoglobulin lambda constant 3
Immunoglobulin lambda variable 1-40
Immunoglobulin lambda variable 1-47
Immunoglobulin lambda variable 1-51
Immunoglobulin lambda variable 2-11
Immunoglobulin lambda variable 2-23
Immunoglobulin lambda variable 2-8
Immunoglobulin lambda variable 3-10
Immunoglobulin lambda variable 3-19
Immunoglobulin lambda variable 3-21
Immunoglobulin lambda variable 3-25
Immunoglobulin lambda variable 3-27
Immunoglobulin lambda variable 3-9
Immunoglobulin lambda variable 5-39
Immunoglobulin lambda variable 5-45
Immunoglobulin lambda variable 6-57
Immunoglobulin lambda variable 7-43
Immunoglobulin lambda variable 7-46
Immunoglobulin lambda variable 8-61
Immunoglobulin lambda variable 9-49
Immunoglobulin lambda-1 light chain
Immunoglobulin lambda-like polypeptide
Immunoglobulin mu heavy chain
2
1
0.5
0
-0.3
-0.5
-1

C

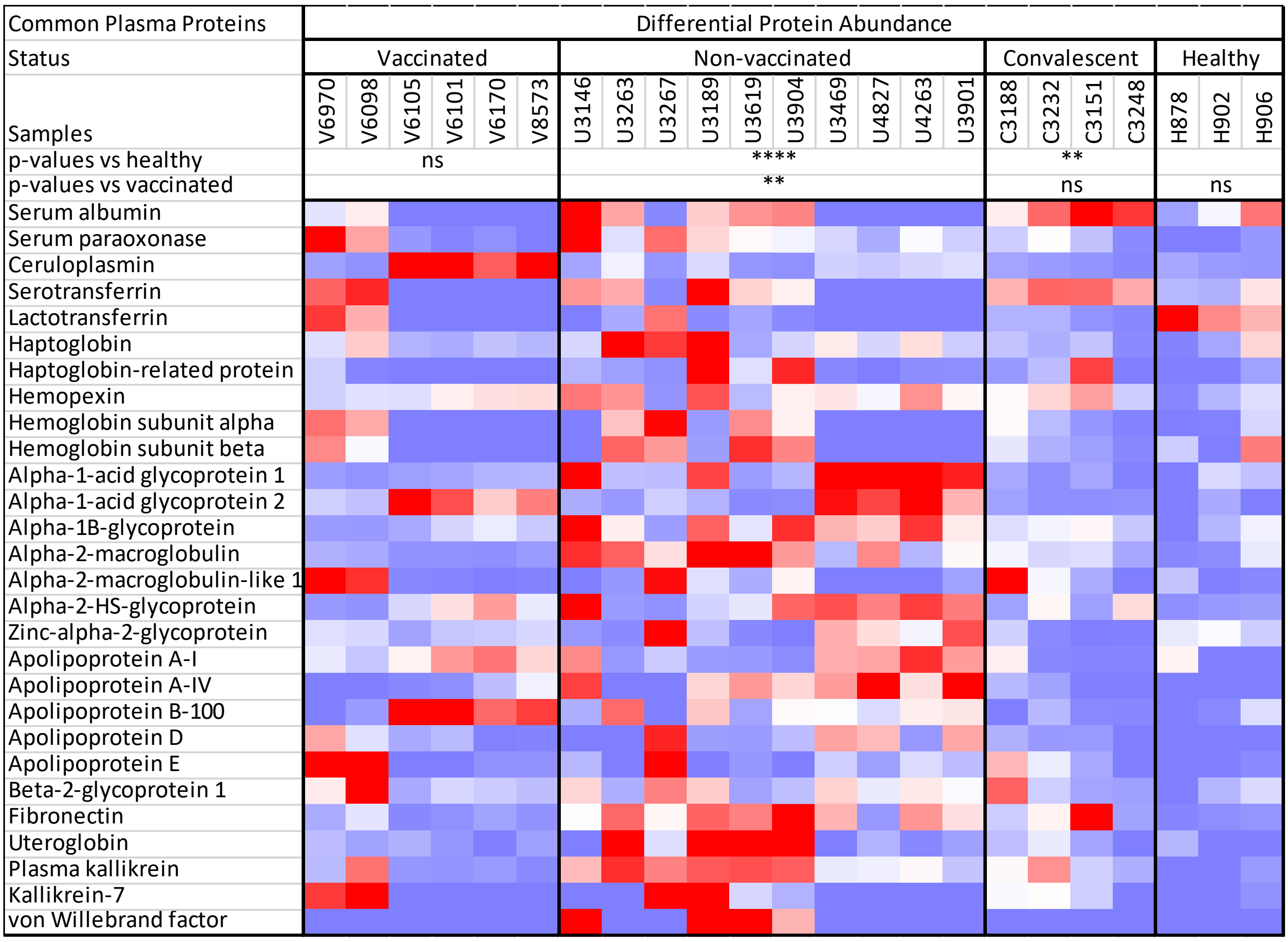

D

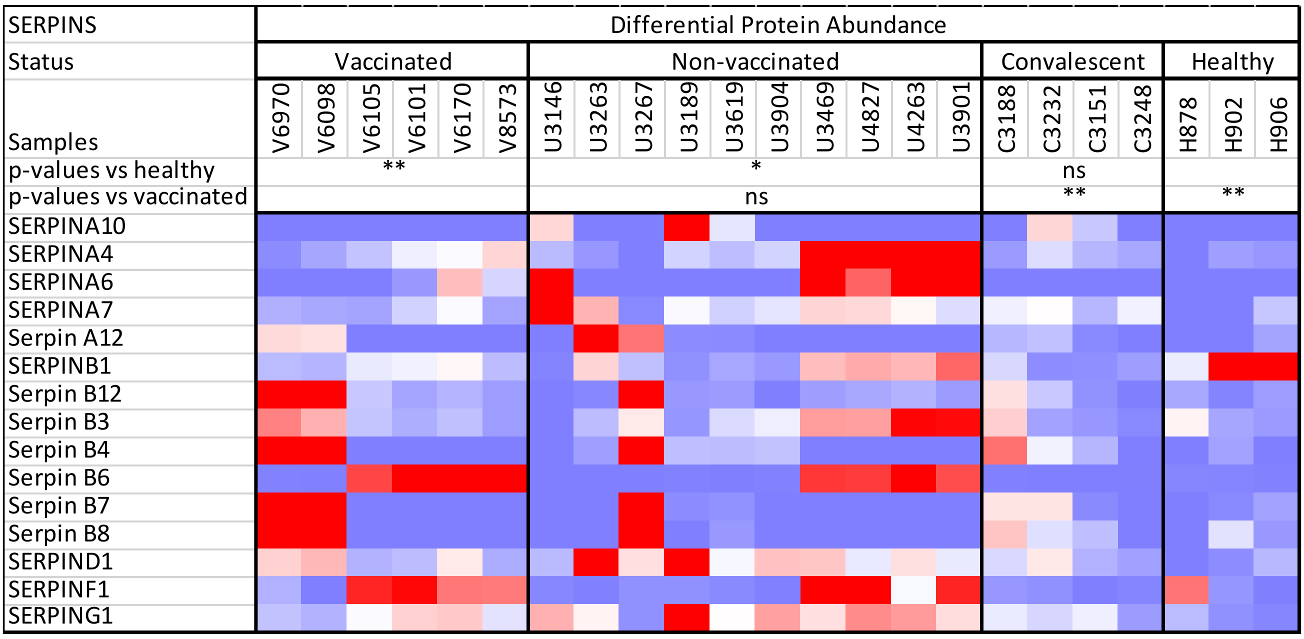

Supplemental Figure 2 Proteomics analyses of proteins in various BALF. A) Heat map showing differential abundances of each category of plasma proteins between acute (vaccinated, non-vaccinated), convalescent COVID and healthy BALF samples. B) List of Immunoglobulins. C) common plasma proteins and D) SERPINS detected in various BALF. Statistical analyses were performed using two-way ANOVA between columns with p-values indicated as *<0.05, **<0.01, ***<0.001, ****<0.0001.

Supplemental Figure 3

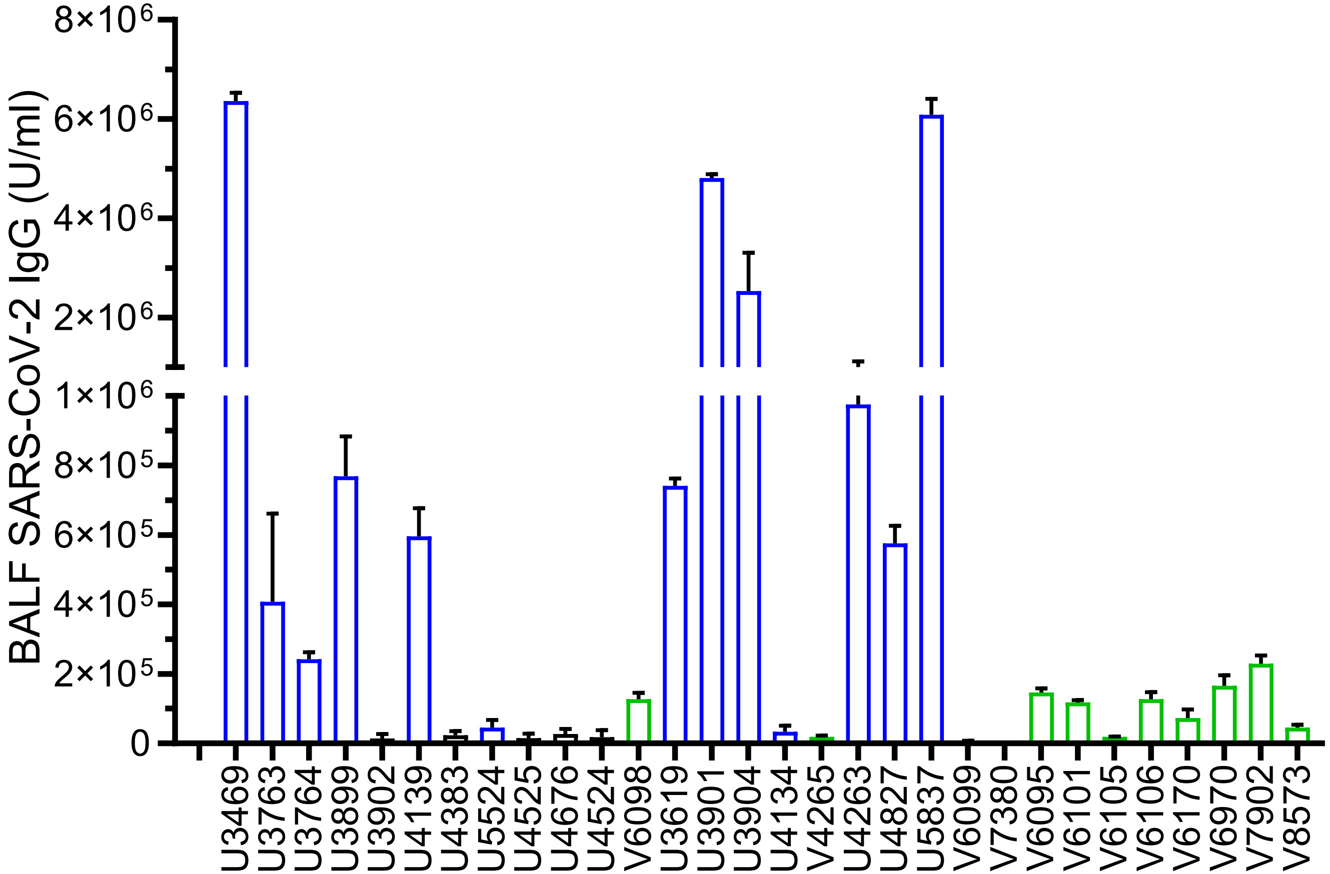

Supplemental Figure 3. The presence of SARS-CoV-2 spike specific IgG in vaccinated (green) and non-vaccinated COVID BALF samples as measured by ELISA.

Supplemental Figure 4

A

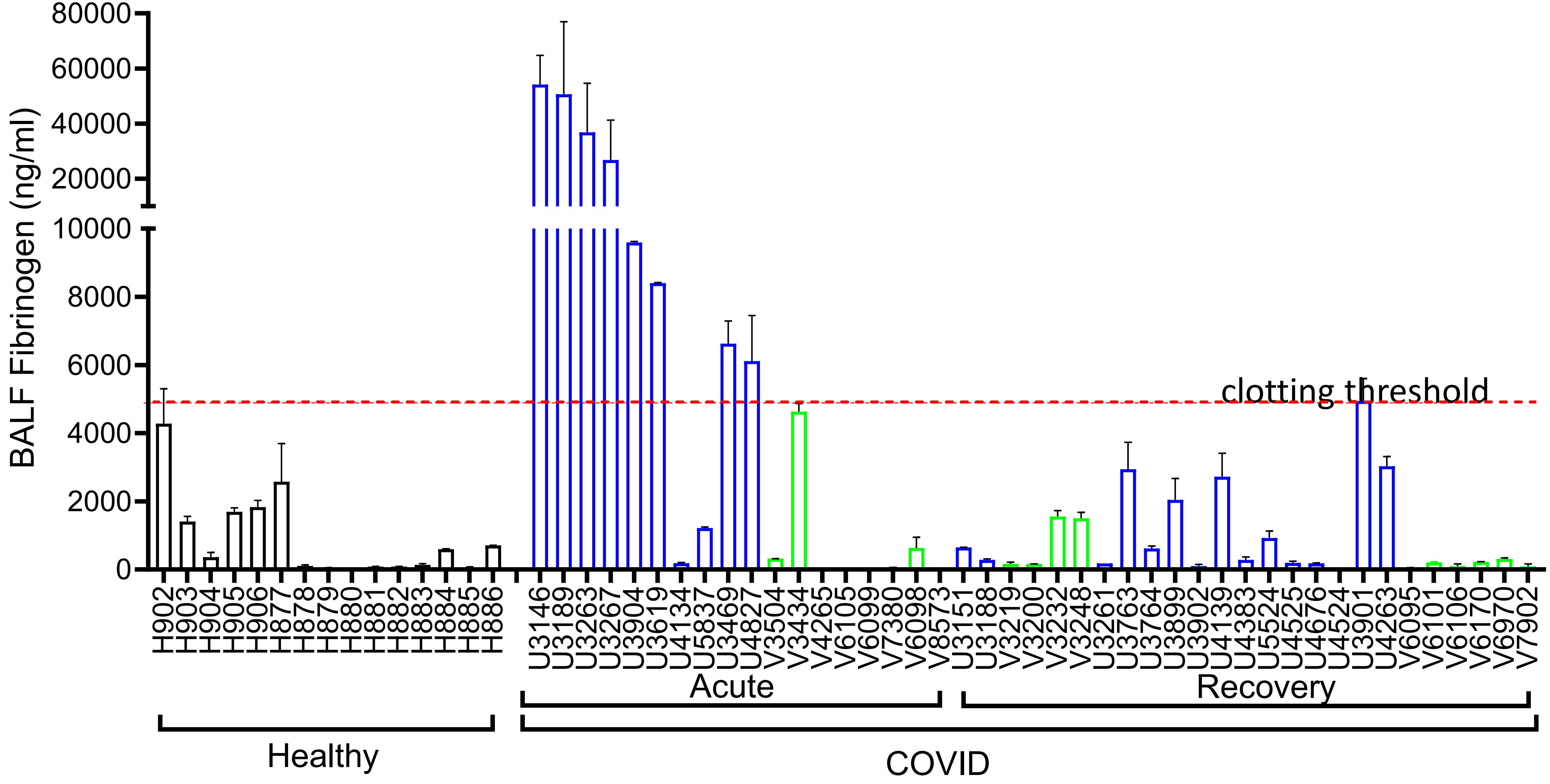

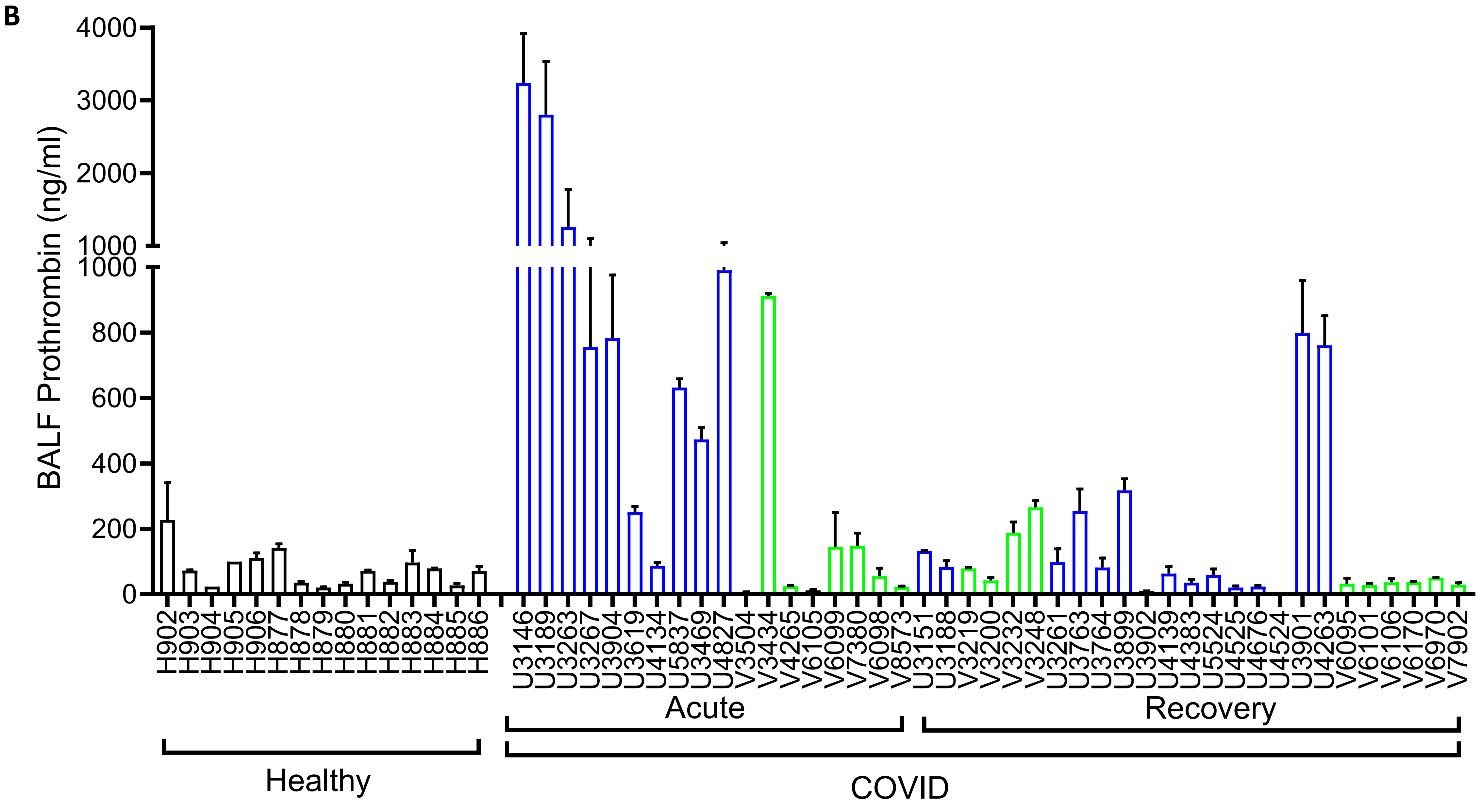
B
BALF Prothrombin (ng/ml)
4000
3000
2000
1000
1000
800
600
400
200
0
H902
H903
H904
H905
H906
H877
H878
H879
H880
H881
H882
H883
H884
H885
H886
U3146
U3189
U3263
U3267
U3904
U3619
U4134
U5837
U3469
U4827
V3504
V3434
V4265
V6105
V6099
V7380
V6098
V8573
U3151
U3188
V3219
V3200
V3232
V3248
U3261
U3763
U3764
U3899
U3902
U4139
U4383
U5524
U4525
U4676
U4524
U3901
U4263
V6095
V6101
V6106
V6170
V6970
V7902
Acute
Recovery
Healthy
COVID

C

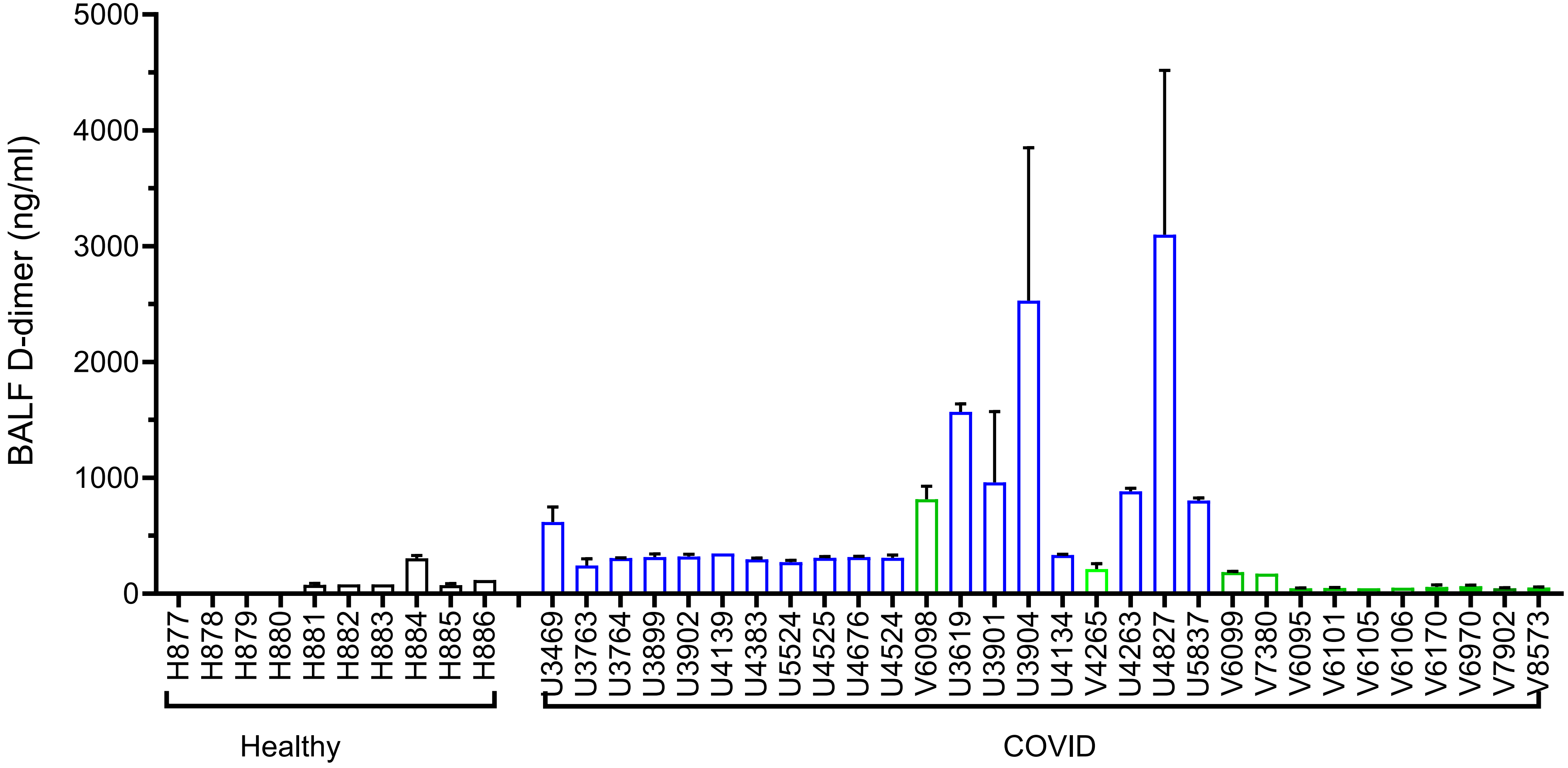

Supplemental Figure 4 ELISA measurement for fibrinogen (A), prothrombin (B) and D-dimer (C) concentrations in Acute, Recovery COVID and healthy BALF samples with vaccinated individuals colored in green and non-vaccinated in blue.

Supplemental Fig 5

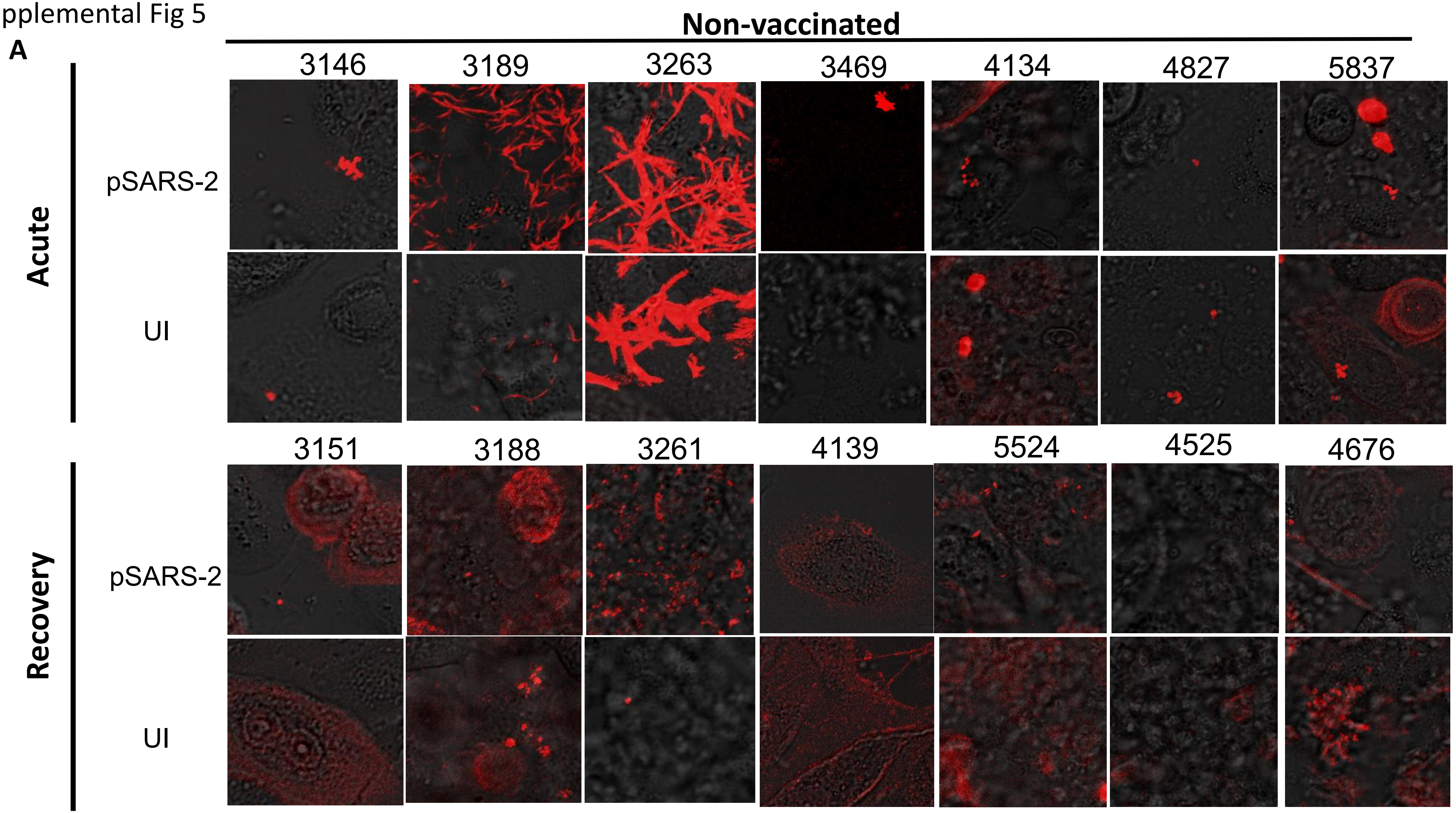

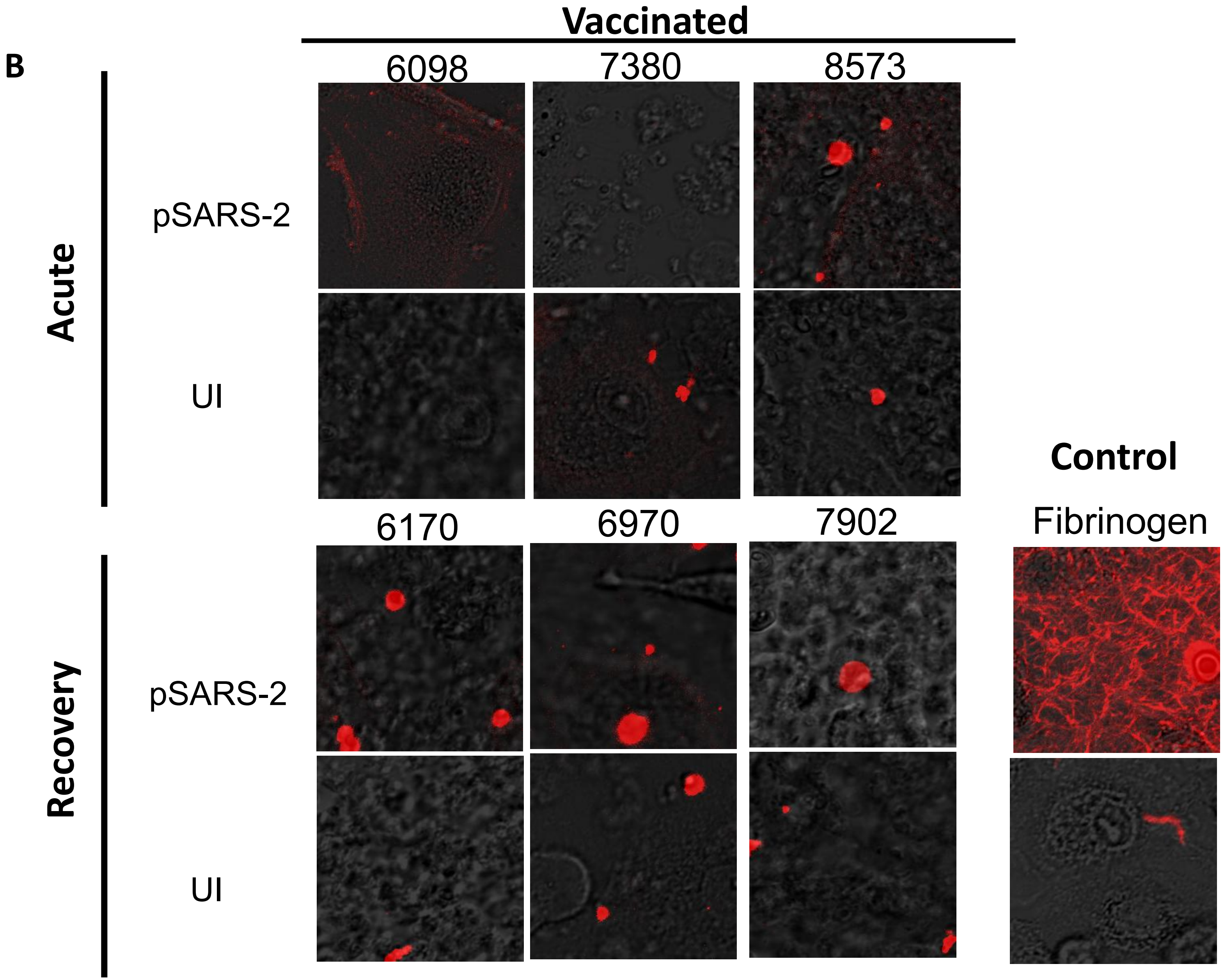

Supplemental Figure 5. SARS-CoV-2 infection induces fibrins in BALF. A-B) Fibrin depositions in non-vaccinated (A) and vaccinated (B) BALF samples. HSAEC cells were infected with pseudo-typed Omicron SARS-CoV-2 viruses (SARS-2) or mock (UI) for 24-48 hours before replacing media with various BALF in the presence of clotting buffer.

Supplemental Figure 6

A

OM (µL) 0.095 0.19 0.38 0.75 1.56 3.12

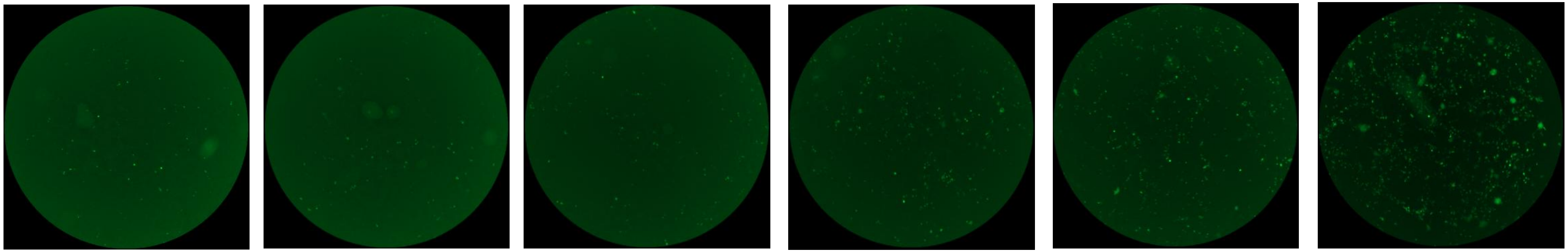

B

Core (µL) 1 2

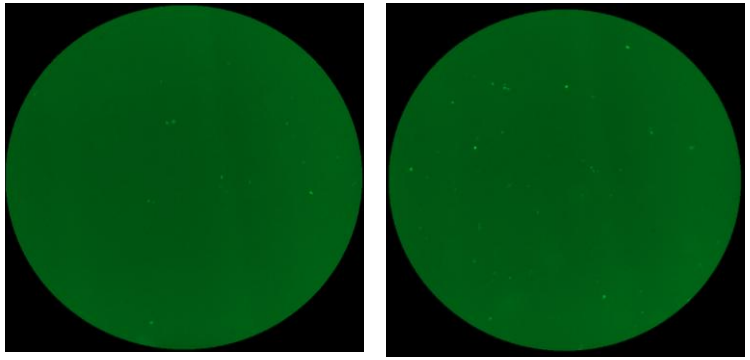

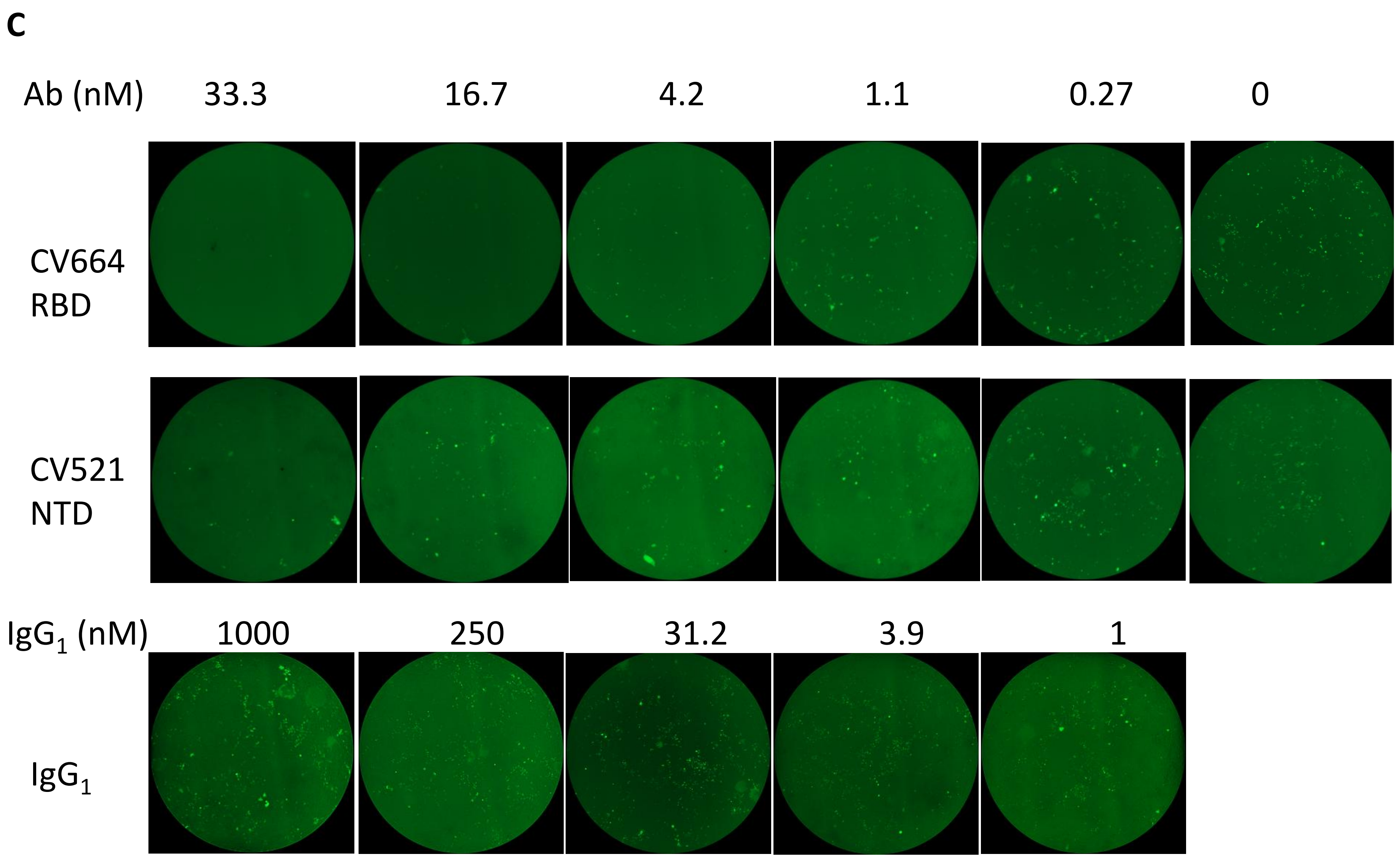

Supplemental Figure 6. SARS-CoV-2 dose-dependent viral infection. A-B) Infection of ACE2-293T cells with varying doses of a GFP-expressing SARS-CoV-2 spike-containing (A) or spike-lacking (B) pseudoviruses in a 384-well plate. One microliter of the pseudovirus results in a 10:1 ratio for copies of viral RNA to number of cells. C) Antibody neutralization of SARS-CoV-2 Wuhan pseudovirus infection of ACE2-293T cells. Titration-dose of neutralizing antibodies, CV664 and CV521, specific for RBD and NTD of SARS-CoV-2, respectively, were added to ACE2-293T cells together with a constant dose of GFP-expressing omicron pSARS-2 viruses. The infections were determined at 48-72 hours post infection as % of GFP+ cells in the presence versus absence of the antibodies. IgG_1 was used as a control for neutralization antibodies.

Supplemental Fig 7

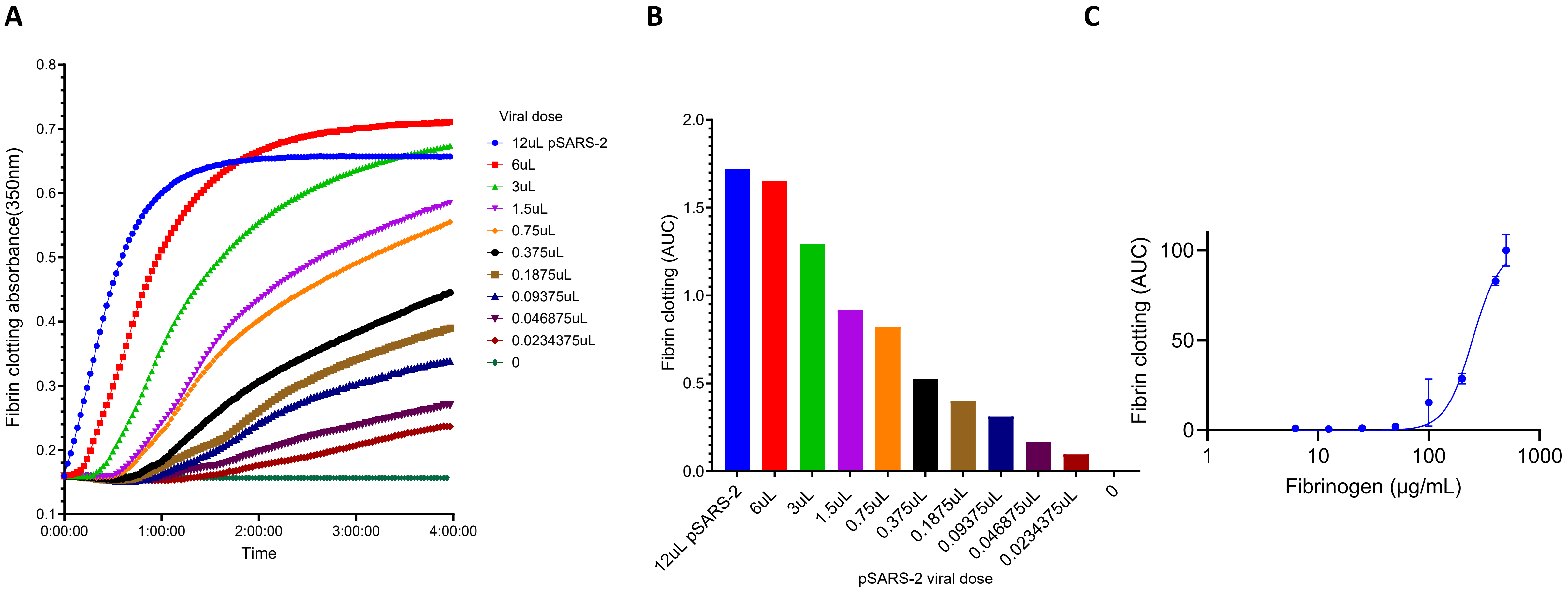

Supplemental Figure 7. Fibrin clotting induced by SARS-CoV-2 infection is proportional to the viral dose and fibrinogen concentration. A-B) Kinetic clotting curves (A) and the extent of fibrin clotting measured as area under the curve (AUC) (B) from pSARS-2 infected NHBE cells in the presence of titrating amount of the virus. C) Dependence of viral-induced fibrin clotting on fibrinogen concentration.
